# Supplementary material for: The development of single-cell lineage tracing technology and its application in immunotherapy
Source: Mol Ther Adv. 2026 Jul 2;34(3):201802. doi: 10.1016/j.omta.2026.201802 (PMC13392950; doi:10.1016/j.omta.2026.201802)
Supplement: Document S1. Tables S1–S3 [file mmc1.pdf]

**OMTA, Volume *34***

## Supplemental information

# The development of single-cell lineage tracing technology and its application in immunotherapy

**Jun Wang, Zhisen Li, Jia Chen, Huan Li, Fei Chen, Lei Tan, Xiaoge Chen, Song Liu, Wenfeng Zhang, and Hongwei Shao**

Table S1: Comparison of core characteristics across different cell lineage tracing barcode types

| Type | Characteristics | Application | Method | Principle | Source Tagging | Testing Method | Advantages | Limitations | References |
|---|---|---|---|---|---|---|---|---|---|
| Exogenous engineered barcodes (Static) | Relies on the integration of synthetic DNA elements; mainly used for clone identification and early expansion analysis | Suitable for experiments requiring high controllability, such as developmental biology and tumor heterogeneity studies | Polylox barcode | Exogenous Cre recombinase mediates stochastic recombination between multiple loxP sites within a preset barcode cassette, generating unique heritable DNA rearrangements | Exogenously introduced loxP site arraysou | Identify rearranged sequences of the exogenous loxP array via singlecell sequencing (scRNAseq/snRNAseq) | Controllable labeling (inducible by light or drugs); high flexibility; generates high barcode diversity | Requires exogenous Cre expression; recombination efficiency is affected by loxP site configuration | Orban et al.41 |
| | Uses transposons or lentiviruses to randomly integrate exogenous DNA fragments; integration sites serve as markers | Suitable for labeling stem cells, cancer cells, and experiments requiring high labeling diversity | Integration barcode | Transposons or lentiviruses randomly integrate exogenous DNA fragments into the host genome; the integration sites or fragments themselves serve as unique barcodes that are inherited through cell divisions | Exogenously integrated DNA fragments | Identify integrated DNA fragments via singlecell sequencing (scRNAseq/snRNAseq) or thirdgeneration sequencing | High marker diversity; applicable to stem cells and cancer cells | Viral integration may affect genomic stability; randomness can lead to uneven distribution of markers | Schmidt et al.42 |
| Exogenous engineered barcodes (Dynamic) | Uses programmable nucleases to induce mutations at specific loci, enabling longterm lineage recording and fate branching analysis | Suitable for simultaneous lineage tracing and singlecell transcriptomics; construction of highresolution lineage trees | LINNAEUS | CRISPR/Cas9 induces insertions and deletions (InDels) at specific genomic loci; these “genetic scars” serve as barcodes read out by scRNAseq | InDels induced by CRISPR/Cas9 | Analyze scar sequences generated by InDels using bulk RNAseq or scRNAseq | Singlecell resolution; compatible with scRNAseq; enables dynamic recording of cellular processes. | Editing efficiency may be limited by the number of available target sites | Spanjaard et al.43 |
| | Similar to LINNAEUS, but built into an engineered mouse model that allows druginducible initiation of recording at any time point | Suitable for highresolution in vivo lineage tracing, especially when recording needs to be initiated at specific time points | CARLIN | An engineered mouse model harboring an array of gRNA targets and Teton inducible Cas9; doxycycline induces Cas9 to generate InDel mutations at the target array, producing transcriptionally compatible barcodes | InDels induced by CRISPR/Cas9 | Analyze InDel scar sequences via bulk RNAseq or scRNAseq | Singlecell resolution; compatible with scRNAseq; dynamically inducible at desired time points | Requires preengineered mouse model; editing efficiency is influenced by the number of available targets | Bowling et al.44; Abdullah et al.45 |
| | Uses a cytidine deaminase to introduce C→T substitutions in a barcode cassette, avoiding doublestrand breaks | Suitable for longterm, lowperturbation lineage recording | SMALT system | A cytidine deaminase (AID) introduces heritable C→T substitutions within an engineered barcode cassette, avoiding doublestrand breaks and reducing information loss | Base substitutions induced by cytidine deaminase | Identify base substitutions via scRNAseq or snRNAseq. | No doublestrand breaks (DSBs), advantageous for monitoring sensitive processes like embryogenesis; highdensity labeling | Relies on exogenous mRNA delivery; requires highthroughput sequencing analysis | Liu et al.46; Chen et al.47 |
| | Uses a modified hgRNA to guide Cas9 to cut its own transcription site, accumulating mutations to form dynamic barcodes | Suitable for cumulative recording systems requiring extremely high barcode diversity | hgRNA/CRISPR | hgRNA guides Cas9 to cut its own transcription site; each cut generates InDels. Combining multiple hgRNA sites exponentially increases barcode diversity | InDels induced by CRISPR/Cas9 | Analyze InDel mutations at hgRNA loci by sequencing | Extremely high barcode diversity; enables cumulative recording. | Depends on delivery and expression of the exogenous system | Kalhor et al.48 |
| Endogenous geneticbarcode (Inducedbarcodes) | Uses base editors to introduce singlenucleotide variants without generating doublestrand breaks | Suitable for longterm lineage tracing, especially when avoiding DSBs is critical | Base editor barcode | Base editors (e.g., dCas9deaminase fusions) introduce precise singlenucleotide variants (SNVs) at target DNA sequences; accumulated mutations serve as heritable barcodes | SNVs induced by base editors | Identify base substitutions via singlecell sequencing | No DSBs, favorable for monitoring sensitive processes such as embryogenesis; highdensity labeling | Depends on delivery of exogenous base editors; requires highthroughput sequencing analysis | Hwang et al.49; Zhao et al.50; Li et al.51 |
| | Utilizes the cell’s own genetic variation (e.g., mitochondrial DNA mutations) for lineage tracing without introducing exogenous sequences | Suitable for most cell types, especially primary cells sensitive to genetic manipulation and clinical samples | Mitochondrial DNA barcode | Uses the inherently high mutation rate of mitochondrial DNA (mtDNA) (10–100× that of nuclear DNA) to naturally accumulate mutations during cell proliferation, forming genetic markers | Natural mutations in mtDNA as endogenous biomarkers | Capture mtDNA sequences and detect mutations via singlecell sequencing (e.g., scRNAseq, scATACseq) | Noninvasive, no gene editing required; suitable for primary cells, especially T cells | mtDNA copy number variation can affect marker reliability; applicability may be limited across different tissue types. | Weng et al.52; Rusk53; Lareau et al.54; Ludwig et al.55 |
| Endogenous geneticbarcode (Naturalbarcodes） | Uses the vast diversity generated by Tcell receptor (TCR) gene rearrangement as a clonal marker, especially suitable for Tcell lineage tracing | Applicable to Tcell lineage tracing; used to study Tcell clonal evolution, tumor microenvironment, and treatment efficacy | TCR barcode | TCR generates an extremely diverse repertoire through somatic recombination. Each T cell and its progeny carry a unique TCR sequence that serves as a lineage marker | TCR gene sequences as natural barcodes | Identify TCR sequences via singlecell TCR sequencing (scTCRseq) or combined scRNAseq | Noninvasive, no gene editing required; suitable for primary T cells; directly reflects Tcell clonal dynamics | TCR sequences are highly heterogeneous with minimal sharing between patients—highly personalized; can only track Tcell clones, not reconstruct complete developmental lineages | Zhang et al.56; Cao et al.57 |

Table S2. Summary of Single-Cell Lineage Tracing and Trajectory Inference Methods

| Method | Main principle | Main use | Reference |
|---|---|---|---|
| Monocle | Orders cells along pseudotime based on transcriptional similarity | Reconstruction of developmental trajectories | Qiu et al. 63 |
| Slingshot | Infers lineage structure using cluster-based minimum spanning trees and simultaneous principal curves | Branching trajectory inference | Street et al.61 |
| TSCAN | Uses clustering and minimum spanning tree-based ordering | Pseudotime analysis | Ji and Ji.62 |

Table S3: Detailed comparison and future perspectives of singlecell lineage tracing (SCLT) strategies

| Strategy | Barcode Type | More Suitable Application Scope | Experimental Complexity | Integration with scRNA-seq | Already Used for T Cell / CAR-T Lineage Tracing | Main Advantages | Main Limitations | Latest Progress and Prospects | References |
|---|---|---|---|---|---|---|---|---|---|
| TCR barcode | Endogenous, clonotype-based | Most directly applicable to T cells/engineered T cells; short- to mid-term clonal expansion, persistence, state differentiation | Moderate | Strong; scRNA + paired TCR-seq is mature | T cells: widely used; CAR-T: clearly used for in vivo clonal dynamics studies in patients | Natural, no labeling required; directly corresponds to antigen recognition history; most suitable for T cell studies | Can only track clones after V(D)J rearrangement; cannot reveal earlier ancestors or intra-clonotype hierarchy | Moving from "clonal counting" toward clonal fate rules and longitudinal efficacy association; one of the most practical barcodes for clinical engineered Tcell studies | Zhang et al.57; Cao et al.58 |
| mtDNA barcode | Endogenous, somatic mutation-based | Short-term tracking of primary cells; clinical cohorts and pathology samples (more accessible than most prospective engineered barcodes) | High (limited by sequencing depth and platform type) | Achieved (e.g., integrated with spatial transcriptomics) | T cells: proven feasible; CAR-T: currently nonmainstream | No exogenous gene introduction; high usability in clinical samples | Limited by mitochondrial inheritance patterns and tissue applicability; detection constrained by sequencing depth/platform; mtDNA variants not strictly neutral in all contexts | Developed to work with frozen samples and spatial transcriptomics for lineage tracing in intact human tissues | Ludwig et al.55; Lareau et al.54; Salla et al.80; Ludwig et al.81 |
| Integration barcode | Exogenous, insertion-based | Immune cell expansion and fate analysis; research models, cell product evaluation, preclinical validation | Moderate (library construction and viral packaging required) | Integrated (e.g., LARRY) | CAR-T: used for clonal dynamics analysis of products (combined with integration site analysis) | Relatively simple to construct; can label large numbers of cells at once | Random integration may affect host gene regulation or cell fitness; insertion site safety must be carefully assessed | Needs optimization for safety and clinical translation; LARRY links scRNAseq state map to cell fate map | Schmidt et al.45; Weinreb et al.74; Hayal et al.75; Sheih et al.76 |
| Polylox barcode | Exogenous, DNA recombination-based (Cre-loxP) | Precise in vivo barcode generation; long-term in vivo tracking; conditional gene switches/circuit control; prebuilt in transgenic mice (not directly applicable to patients) | High (requires Cre-loxP system and transgenic mouse models) | Achieved (PolyloxExpress allows simultaneous barcode + transcriptome reading) | T cells: rarely used, not a mainstream strategy | Does not depend on cellular transcriptional expression; suitable for long-term in vivo tracking; enables high-resolution endogenous labeling in vivo | Barcode diversity determined by preset cassette structure; cannot be directly applied to patients | Requires further development for use in T cells; already applied to HSC fate mapping | Orban et al.44; Pei et al.78; Pei et al.79 |
| SMALT (substitution-based) | Exogenous, basesubstitution (AID) | Tracking cell division and clonal expansion history (e.g., addressing whether CART expansion is dominated by few clones or many) | High (requires expression of AID, etc.) | Potential, but no mature solution mentioned | Not explicitly mentioned, but inferred to be applicable for CART clonal expansion studies | Less prone to information loss from large deletions; better for long-term recording; substitution patterns resemble SNP data, allowing use of phylogenetic frameworks | Not uniformly random; sequencecontext bias (e.g., WRC hotspots); risk of convergent edits and homoplasy; writing rate not an intrinsic clock | Has potential to resolve postinfusion CART clonal dominance and longterm persistence | Liu et al.46; Chen et al.47; Pham et al.72; Martin and Scharff73 |
| Base editor barcode | Exogenous, baseediting (nCas9/deaminase) | Similar to SMALT, suitable for continuous mutation recording | High (requires nCas9/deaminase system) | Challenging; requires deep sequencing and rational barcode design | Not explicitly mentioned; early stage | Does not rely on doublestrand breaks; avoids large deletions and target collapse; allows continuous substitution mutations | Writing rate not synchronized with mitosis; not fully random; influenced by editable motif distribution, deaminase sequence bias, and narrow editing window; immature technology | Needs further research and optimization; Transformer Base Editor achieves up to 90% editing efficiency in primary human T cells | Hwang et al.49; Zhao et al.50; Li et al.51 |
| LINNAEUS (CRISPR scar) | Exogenous, CRISPR indelbased | Prospective, hierarchical preclinical studies (reconstructs lineage trees but rarely captures all division events) | High (requires transgenic reporter sites and inducible Cas9) | Integrated (scar barcodes read out by scRNAseq) | Has potential, but limited by editingassociated toxicity and impact on T cell function | Inducible, prospective, controllable | Scar generation not directly linked to mitosis; barcode generation stops early, leading to incomplete coverage of terminal branches; indel bias, homoplasy, dropout, etc. | Dropout can be corrected by introducing "barcode+UMI+scar sequence"; methods like moslin integrate lineage constraints into state alignment | Spanjaard et al.41; Mao et al.65; Lange et al.66 |
| CARLIN / DARLIN | Exogenous, CRISPR indelbased (DARLIN adds TDT) | Prospective, hierarchical preclinical studies; DARLIN suitable for applications requiring high barcode diversity (e.g., in mice) | Very high (requires engineered mouse models or complex constructs) | Integrated (DARLIN enables multiomics like Camelliaseq) | Mostly limited to mouse models; CARLIN used for lineage tracing of antigenspecific CD8 T cell clones | Inducible, generalizable; DARLIN has larger barcode capacity and diversity (via TDT); supports multiomics | Same issues as other indel barcodes (bias, homoplasy, dropout, low recovery rates); editingassociated genotoxicity, offtarget effects; potential impact on T cell fitness | Camelliaseq enables fourmodal singlecell profiling (barcode, transcriptome, chromatin accessibility, DNA methylation) but requires deep coverage and is costly | Bowling +A1:J10et al.42; Abdullah et al.43; Li et al.67 |
