# Supplementary material for: The development of single-cell lineage tracing technology and its application in immunotherapy
Source: Mol Ther Adv. 2026 Jul 2;34(3):201802. doi: 10.1016/j.omta.2026.201802 (PMC13392950; doi:10.1016/j.omta.2026.201802)
Supplement: Document S2. Article plus supplemental information [file mmc2.pdf]

Molecular Therapy
Advances
Review

The development of single-cell lineage tracing technology and its application in immunotherapy

Jun Wang,[1] Zhisen Li,[1] Jia Chen,[1] Huan Li,[1] Fei Chen,[2] Lei Tan,[1] Xiaoge Chen,[1] Song Liu,[3] Wenfeng Zhang,[1] and Hongwei Shao[1]

[1]School of Life Sciences and Bio-pharmaceutics, the First Affiliated Hospital, Guangdong Pharmaceutical University, Guangzhou, People's Republic of China; [2]Xianning Traditional Chinese Medicine Hospital, Xianning, People's Republic of China; [3]School of Pharmacy & Clinical Pharmacy, Guangdong Pharmaceutical University, Guangzhou, People's Republic of China

Immunotherapy stands as one of the most promising approaches in cancer treatment, with engineered T cell therapies, particularly chimeric antigen receptor T cell (CAR-T), leading the charge. However, relapse in some patients post-treatment suggests that research in this field remains incomplete. Tumor heterogeneity and the complexities of the immune microenvironment hinder a comprehensive understanding of the changes engineered T cells undergo once introduced into the human body. Single-cell lineage tracing (SCLT) technology facilitates the investigation of cellular development by monitoring the fate and differentiation of individual cells and their descendants within an organism. Employing methodologies such as CRISPR-based labeling and mitochondrial DNA tracking, SCLT allows for dynamic analysis of T cell clonal evolution, exhaustion mechanisms, and memory cell generation. This approach offers single-cell resolution data that contribute to resolving pertinent clinical challenges. This article provides a comprehensive review of recent developments and characteristics of the SCLT multi-omics approach. It elucidates the manner in which SCLT addresses the conventional constraints associated with spatiotemporal resolution and introduces a novel methodology for generating DNA barcodes to monitor CAR-T cells via CRISPR technology. These contributions offer valuable perspectives for the enhancement of cell therapy strategies.

INTRODUCTION

Although early clinical studies of chimeric antigen receptor T (CAR-T) cells were reported in the early 21st century, including trials in HIV infection[1] and solid tumors,[2] the field achieved a major clinical breakthrough in 2011, when Porter and colleagues reported remarkable efficacy of CD19-targeted CAR-T cells in chronic lymphocytic leukemia.[3] By 2024, ten CAR-T therapies have been developed for various hematological malignancies.[4–8] A search of ClinicalTrials.gov reveals 966 ongoing CAR-T treatment studies in China alone (https://clinicaltrials.gov/). CAR-T therapy, a form of adoptive immune cell therapy, involves a chimeric molecule that consists of an antigen recognition domain, a hinge region, a transmembrane domain, and an intracellular signaling domain. The extracellular domain typically consists of a single-chain variable fragment (scFv) that specifically recognizes tumor-associated surface antigens, whereas the intracellular region usually contains a T cell receptor (TCR)-associated signaling domain, most commonly CD3ζ, together with one or more co-stimulatory domains such as CD28 or 4-1BB.[2] The CAR gene is introduced into the patient's T cells, enabling them to express receptors that target specific antigens on tumor cell surfaces.[9–11] This mechanism allows for selective tumor cell eradication without damaging normal cells, aiming for cancer remission. A key advantage of CARs is their versatility, as they can recognize not only proteins but also carbohydrates and glycosphingolipid structures, broadening the range of potential targets while minimizing harm to healthy cells. Additionally, CARs can target tumor cells with reduced human leukocyte antigen (HLA) expression or defects in proteasomal antigen processing, both of which allow tumors to evade TCR-mediated immunity.

Although the design of CAR molecules offers T cells enhanced targeting versatility, physical barriers within the solid tumor microenvironment, such as dense stroma, and immunosuppressive factors like PD-L1, substantially impair the infiltration and persistence of CAR-T cells. Additionally, CAR-T therapy can trigger immune-related adverse events (IRAEs),[12] with the severity and variety of these events varying due to tumor heterogeneity. Reports have documented cases of relapse post-remission, as well as adverse effects, such as cytokine release syndrome (CRS) and neurotoxicity during

https://doi.org/10.1016/j.omta.2026.201802.

Correspondence: Song Liu, School of Pharmacy & Clinical Pharmacy, Guangdong Pharmaceutical University, Guangzhou, People's Republic of China.
E-mail: songliu@gdpu.edu.cn
Correspondence: Wenfeng Zhang, School of Life Sciences and Bio-pharmaceutics, the First Affiliated Hospital, Guangdong Pharmaceutical University, Guangzhou, People's Republic of China.
E-mail: zhangwenfeng@gdpu.edu.cn
Correspondence: Hongwei Shao, School of Life Sciences and Bio-pharmaceutics, the First Affiliated Hospital, Guangdong Pharmaceutical University, Guangzhou, People's Republic of China.
E-mail: shaohongwei@gdpu.edu.cn

Published by Elsevier Inc. on behalf of The American Society of Gene and Cell Therapy.
This is an open access article under the CC BY license (http://creativecommons.org/licenses/by/4.0/).

treatment.[2] Approximately 30% of patients with lymphoma undergoing CAR-T therapy experience immune effector cell-related neurotoxicity syndrome (ICANS).[13] Importantly, the mechanisms underlying treatment-related adverse events, including IRAEs and CRS, are closely tied to the excessive activation and clonal expansion heterogeneity of engineered T cells, underscoring the need for *in vivo* tracking of T cell dynamics. Although early approaches[14,15] have provided foundational insights into lineage tracing, they are hindered by limitations in the stability and resolution of lineage markers, making them insufficient for the complex demands of biological analysis. Leveraging the advanced analytical capabilities of single-cell technology, single-cell lineage tracing (SCLT) has emerged as a critical tool for studying the dynamic behavior of engineered T cells. By integrating genomic markers with multi-omics data, SCLT can elucidate both the clonal origins and functional evolution of these cells. Thus, the *in vivo* analysis of CAR-T cell behavior has become pivotal in optimizing therapeutic strategies, with SCLT offering a high-resolution solution to this challenge.

Despite the remarkable clinical success of CAR-T-cell therapy, major challenges remain, including heterogeneous expansion, limited persistence, treatment resistance, relapse, and variable differentiation outcomes across infused clones. These unresolved questions make engineered T cell therapy a particularly compelling context in which to apply single-cell lineage-tracing approaches. In this review, we focus on lineage tracing and barcoding strategies that have been or can be readily applied to engineered T cell systems within the field of immuno-oncology, particularly CAR-T and TCR-T therapies. This article does not aim to provide a comprehensive overview of all lineage tracing techniques; rather, it emphasizes the methods most relevant to key issues, such as the dynamics of engineered T cell clones, cell state transitions, persistence, dysfunction, and therapeutic responses.

THE DEVELOPMENT OF CELL LINEAGE TRACING

Various methods have been used for lineage tracing. Early studies relied on direct observation and lineage mapping in embryos,[16,17] while later approaches used labels, such as isotopes, fluorescent proteins, and other tracers to follow cell behavior and developmental origin.[18–22] However, these methods are limited by technical difficulty in labeling small cells and by signal dilution over time, as the markers are not stably inherited.

For effective lineage tracing, markers should satisfy several commonly accepted criteria[23]: (1) biocompatibility: markers should not harm cells or affect development. (2) Heritability/Retention: they should label progenitor cells or entire lineages. (3) Detectability: markers should be observable under an imaging or sequencing through staining or fluorescence. (4) Specificity: markers should bind to or interact with specific cellular substances. (5) Stability: they should provide consistent lineage signals over time.

The development of sequencing technologies has provided new opportunities for the design and detection of lineage markers. In particular, the emergence of strategies based on DNA barcodes enables lineage information to be recorded and retrieved at a much higher resolution than conventional imaging methods. For example, heritable barcode sequences can be introduced into the genome via lentiviral or retroviral integration, allowing clonal relationships to be inferred through sequencing. However, these methods also have limitations. Retroviral vectors often preferentially integrate into specific regions of the genome,[24]The integration site may affect whether the inserted barcode can be effectively detected.[25] To overcome this limitation, researchers developed a highly sensitive polymerase chain reaction (PCR)-based method for lineage tracing of mouse hematopoietic stem cells using integrated barcodes. This strategy combines whole-genome amplification, three-arm ligation-mediated PCR, and next-generation sequencing, enabling the detection of clonal populations at frequencies as low as 5 cells per 10,000 cells.[26]

Further development of single-cell sequencing technologies has greatly expanded the scope of lineage tracing applications. One of the early milestones in single-cell sequencing was the work by Tang and colleagues, who performed single-cell whole-transcriptome mRNA sequencing using the early next-generation sequencing platform SOLiD, which was based on ligase-based sequencing chemistry. This work provided the first systematic characterization of transcriptome expression profiles at the single-cell level.[27] With the continuous improvement of high-throughput sequencing platforms, single-cell amplification methods, and library construction strategies, single-cell sequencing has rapidly expanded from transcriptome analysis to genomics, epigenomics, proteomics, and multi-omics analysis.

At the single-cell genomic level, single-cell genome sequencing enables researchers to resolve tumor clonal architecture, infer clonal evolution, and identify genetic heterogeneity within complex tissues. Improvements in whole-genome amplification protocols have further enhanced genome coverage and variant detection sensitivity.[28] At the single-cell surface proteome level, technologies such as cellular indexing of transcriptomes and epitopes by sequencing (CITE-seq) enable the simultaneous measurement of the transcriptome and cell-surface proteins in individual cells. Building on these advances, integrative single-cell multi-omics has gradually emerged as an important research paradigm, providing a systematic framework for linking molecular states across different biological layers and driving the transition from descriptive analysis of cellular states to mechanistic interpretation.[29]

More importantly, these technological advances have enabled the integration of heritable barcodes or molecular recorders with single-cell omics readouts. This integration allows clonal history, molecular states, and fate trajectories to be linked within the same experiment.[30] In immunotherapy research, single-cell technologies have greatly improved the characterization of immune cell heterogeneity within the tumor microenvironment.[31–34] However, static single-cell profiling alone cannot fully explain how specific immune cell states arise, persist, or evolve over time.

Single-cell lineage tracing (SCLT) addresses this limitation by linking cell state to clonal origin and developmental history, thereby providing a more powerful framework for understanding immune cell fate dynamics and functional heterogeneity in engineered T cell therapies.[35]

In addition to barcode-based sequencing methods, several lineage tracing strategies have been integrated with single-cell omics to study cell fate decisions in specific biological contexts. Zhang and colleagues employed the Cre-loxP system combined with fluorescence protein-based lineage markers to track cells in the perirenal adipose tissue of mice.[36] By performing single-nucleus RNA sequencing at different developmental stages, they revealed dynamic cellular changes during postnatal development. Similarly, Gaublomme and colleagues developed a nucleus hashing strategy, in which nuclei from different samples were labeled with sample barcoding antibodies targeting the nuclear pore complex prior to pooling and demultiplexing for single-nucleus RNA sequencing.[37] Buenrostro and colleagues developed single-cell transposase-accessible chromatin sequencing (scATAC-seq), a scalable method for profiling chromatin accessibility at the single-cell level.[38] Because scATAC-seq preferentially captures DNA fragments from open chromatin regions, it provides a useful modality for linking chromatin accessibility to cell identity and, in certain experimental designs, to lineage barcode information[39].

Together, these technological advances have transformed lineage tracing from a primarily observation-based and labeling-based method into a sequencing-enabled framework with single-cell resolution. Modern SCLT not only reconstructs clonal relationships but also connects lineage history to transcriptional states, chromatin accessibility, surface protein expression, and functional cell fates. This transformation provides the methodological foundation for using SCLT to study development, cancer evolution, immune cell dynamics, and engineered cell therapies.

STRATEGIES FOR LINEAGE BARCODE GENERATION

Different lineage-tracing modalities are suited to different questions in CAR-T/TCR-T therapy. Synthetic inserted barcodes are useful in preclinical models for studying clonal competition, expansion kinetics, and manufacturing bottlenecks,[25,40] whereas editing-based evolving barcodes are better suited for reconstructing lineage branching and state transitions associated with persistence or exhaustion. Endogenous barcodes, especially mitochondrial DNA (mtDNA) variants and TCR clonotypes, are more applicable to patient-derived samples because they are less perturbative and compatible with routine single-cell profiling.[41–43] Therefore, the choice of tracing modality should be matched to the biological and clinical question being investigated.

As illustrated in Table S1, based on the source of the barcodes and the mechanisms by which lineage information is recorded, these strategies can be broadly classified into four categories: exogenous editing-based barcodes, exogenous insertion-based barcodes, endogenous artificially induced barcodes, and endogenous natural barcodes. For engineered T cell therapy, barcode selection should consider not only the biological question being addressed but also manufacturing compatibility, labeling complexity, and safety. In general, the applicability and utility of various SCLT methodologies in engineered T cell therapy differ accordingly. Exogenous dynamic recording systems are particularly well suited for prospective, hierarchical preclinical investigations, whereas endogenous barcoding approaches are more appropriate for analyses involving patient-derived samples and clinically relevant settings.

Exogenous engineered barcodes

Exogenous engineered barcodes denote lineage tracing systems that are artificially incorporated into cells prior to experimentation via viral vectors, transgenic constructs, or gene-editing techniques. Based on the modality of information encoding, these systems can be categorized into static and dynamic barcodes. Static barcodes are primarily employed for clone identification and the analysis of early cellular expansion, whereas dynamic barcodes are better suited for long-term lineage reconstruction and the examination of fate branching.

Static barcodes

Polylox barcode

As illustrated in the Figure 1, Polylox is a SCLT strategy based on Cre/loxP-mediated DNA recombination. In this system, Cre recombinase, derived from bacteriophage P1, recognizes *loxP* sites and catalyzes recombination between them.[44] A *loxP* site is a 34-bp sequence composed of two inverted repeats flanking an asymmetric core sequence. When multiple *loxP* sites are arranged within a barcode cassette, Cre-mediated recombination stochastically generates diverse DNA rearrangements, producing unique heritable barcodes in individual cells. Because Cre activity can be induced in a temporal- or tissue-specific manner, Polylox enables controlled lineage labeling and subsequent reconstruction of clonal relationships.[44] Polylox relies on Cre/*loxP* recombination to generate recombined barcodes within a preset barcode cassette, and it can reactivate barcode generation at specific times and in specific cell populations.

Integration barcode

As illustrated in the Figure 2, the integration barcode system is a SCLT technology that labels cells by integrating short DNA fragments into the genome. This is achieved through the use of transposons or lentiviruses, which randomly integrate these fragments into the genome.[45] The randomly distributed DNA fragments in the host genome serve as barcodes, which are inherited by daughter cells as progenitor cells proliferate and differentiate, thus enabling the tracking of cell fate. The random integration of these fragments provides a diverse array of barcode labels, making it suitable for labeling stem cells and cancer cells. The barcodes generated by integration sites do not rely on the sequence diversity of DNA fragments;

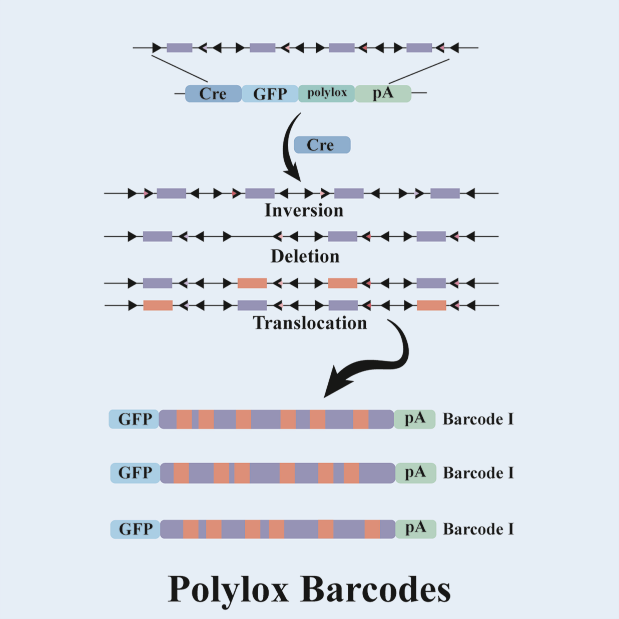

Figure 1. Schematic diagram of the Polylox barcode system generating diverse DNA barcodes through Cre/*loxP* recombination

System construct: The diagram shows the structure of the Polylox reporter construct, which includes a green fluorescent protein (GFP) reporter gene driven by a ubiquitous promoter. Downstream of GFP, a "Polylox" barcode array containing multiple variant *loxP* sites (lox66, lox71, lox2272, etc.) is inserted, followed by a polyadenylation signal (pA). Barcode generation: Upon expression of Cre recombinase (e.g., induced by a cell-type-specific promoter or a tamoxifen/light-controlled system), it recognizes and catalyzes irreversible recombination events (such as deletion, inversion, or exchange) between these *loxP* sites. Each recombination event permanently alters the DNA sequence of the barcode array. Diverse barcode outcomes: Due to the stochastic nature of recombination and the orientation of the *loxP* sites, clonally related cells that initially share the same barcode will accumulate unique and heritable DNA sequence variations after multiple rounds of cell division and Cre-mediated recombination. This process generates a vast diversity of distinct barcodes (as shown in "Barcode I/II/III"), enabling high-resolution lineage tracing.

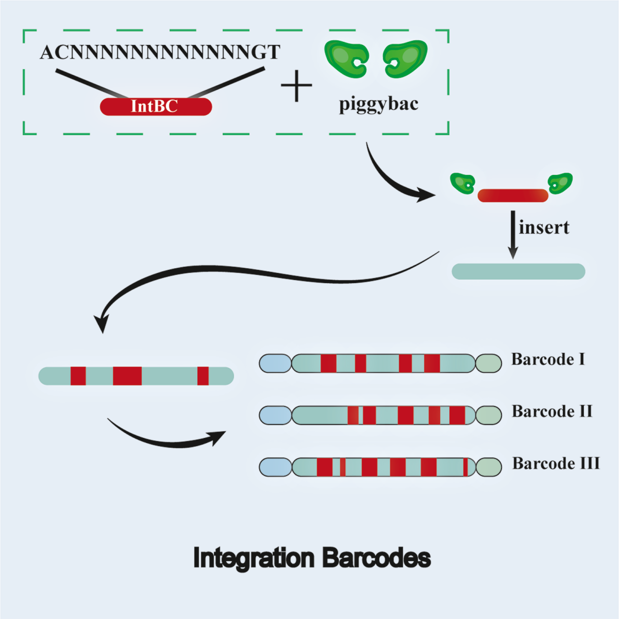

Figure 2. Schematic diagram of DNA barcode system based on transposon integration

The diagram illustrates the core mechanism of the integration barcode system. This system utilizes transposons (e.g., piggyBac) or viral vectors to randomly insert short DNA barcodes containing random sequences (e.g., "ACNNNNNNNNNNNNNT", where N represents random bases) into various locations of the host genome. Each integration event creates a unique and heritable DNA "tag". As progenitor cells carrying different barcodes proliferate and differentiate, these unique integration site sequences are passed on to all progeny cells, serving as permanent lineage marks. The lineage relationships and clonal evolution can then be reconstructed by sequencing and comparing these barcodes.

instead, they use the sites where exogenous fragments integrate into the host genome as barcode diversity. Therefore, they can be considered Inserted DNA barcodes.

Dynamic barcodes

LINNAEUS

As shown in the Figure 3, LINNAEUS (lineage tracing by nuclease-activated editing of ubiquitous sequences) is a method for simultaneously performing lineage tracing and single-cell transcriptome profiling *in vivo*. It uses CRISPR-Cas9-induced genetic scars as barcodes, which are then readout by scRNA-seq.[46] The core of this method is that the CRISPR-generated scars are transcripts detectable by scRNA-seq; by incorporating these scars into the single-cell expression profiles, it can more directly reveal relationships between cells. Currently, CRISPR-Cas9 technology and single-cell transcriptome sequencing are relatively mature, with a relatively low experimental threshold, allowing the preparation of lineage barcodes for a large number of cells.

CARLIN

As illustrated in the Figure 3, closely related to LINNAEUS, CARLIN (CRISPR array repair lineage-tracing with indel mutations) represents a further development of CRISPR-Cas9-based insertions or deletions (indel) lineage recording. In this system, multiple Cas9-target sites are arranged as a barcode array within the 3′ untranslated region (3′ UTR) of a fluorescent reporter transcript. CARLIN is an engineered mouse model that integrates 10 gRNAs, the corresponding target site array (CARLIN array), and Tet-on Cas9 into the genome, allowing recording to be initiated at any given time point using doxycycline. CRISPR-Cas9 is then used to edit these targets, generating indels that serve as lineage markers.[47] This system can be activated at various time points, producing a large number of transcriptional barcodes, making it suitable for high-throughput scRNA sequencing. By identifying CRISPR-Cas-induced indel lineage markers, it is possible to determine cell clone frequencies, map cell development, and investigate cell behavior.[48] CARLIN has already been used for lineage tracing of antigen-specific CD8 T cell clones, demonstrating its suitability for studying *in vivo* clonal expansion and fate differentiation.[48]

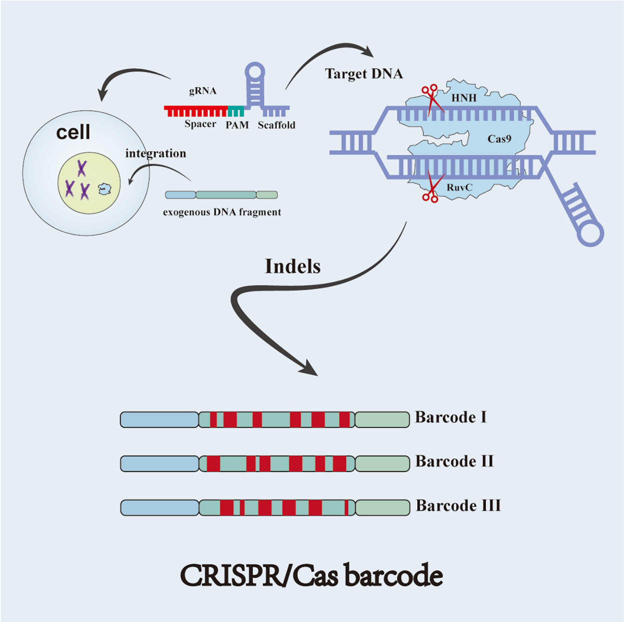

Figure 3. Schematic diagram of DNA lineage barcode generation via continuous CRISPR-Cas9 editing

The core mechanism of lineage tracing using the CRISPR-Cas9 system relies on inducing heritable DNA sequence variations. When a guide RNA targeting a specific genomic locus (target DNA) forms a complex with the Cas9 protein, it creates a DNA double-strand break (DSB) at the target site. These barcode sequences can be recovered together with single-cell transcriptomic or multi-omic readouts, enabling the reconstruction of clonal relationships and the association of lineage history with cell-state transitions.

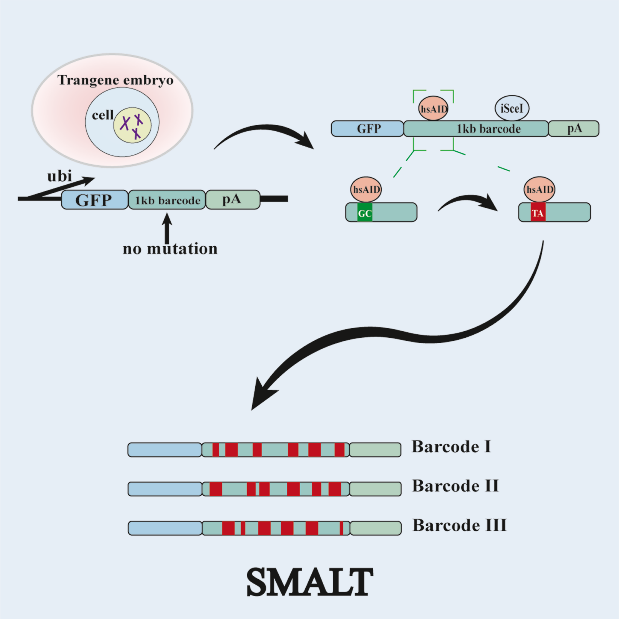

Figure 4. Schematic diagram of the SMALT system

This diagram illustrates the SMALT (substitution mutation-aided lineage-tracing) system applied in zebrafish embryos. The injection of *in vitro* transcribed AID$_{10}$-iSceI-UGI mRNA leads to sustained expression of the fusion protein driven by the ubiquitin promoter (UBI). Green fluorescent protein (GFP) serves as a reporter. The system targets a pre-integrated 1-kb DNA barcode region containing multiple iSceI binding sites (marked in red). Guided by iSceI, the activation-induced cytidine deaminase (AID) induces continuous C-to-T substitution mutations near these sites (accumulated black mutation marks shown in "Barcode I/II/III"), thereby generating unique mutational barcodes for high-resolution lineage tracing.

SMALT system

As illustrated in the Figure 4, SMALT (substitution mutation-aided lineage tracing) is a substitution-based lineage recorder that leverages an activation-induced cytidine deaminase (AID) to introduce heritable C→T (and complementary G→A) substitutions within engineered barcode cassettes, enabling progressive accumulation of lineage marks over time.[49] In contrast, many CRISPR indel recorders rely on Cas9-induced double-strand breaks (DSBs) followed by non-homologous end joining (NHEJ), which yields heterogeneous and biased repair outcomes and can generate inter-target deletions that cause sequence dropout and erase previously recorded information.[50] SMALT enriches single-base substitutions within the barcode region, supporting continuous "writing" with reduced risk of DSB-driven information loss, and is therefore well suited for long-term recording compared with cumulative CRISPR indel barcodes.[49,50]

hgRNA/CRISPR

As illustrated in the Figure 5, homing guide RNA (hgRNA) is a modified version of canonical single guide RNA (sgRNA). By appending a protospacer adjacent motif (PAM) site after the spacer sequence, the hgRNA enables the Cas9-gRNA complex to cleave the hgRNA-encoding sequence itself. Through NHEJ-mediated repair, new hgRNA sequences are generated, allowing the hgRNA to continuously diversify and self-renew. Over time, each hgRNA locus accumulates indels, forming heritable dynamic barcodes.[51] When multiple independent hgRNA sites are introduced into a cell, the diversity of barcodes increases exponentially, greatly enhancing barcode diversity. Therefore, hgRNA/CRISPR functions as an accumulative barcode system.

Endogenous genetic barcode

Endogenous genetic barcodes denote inherent genetic markers that arise through the natural processes of cellular development, division, and the progressive accumulation of mutations. These markers enable the inference of clonal relationships without necessitating the introduction of exogenous barcodes. A primary advantage of this approach lies in its avoidance of supplementary genetic engineering, rendering it particularly applicable to patient-derived specimens and research with clinical relevance.

Endogenous inducible barcode

It refers to lineage markers artificially induced on the native genome of cells by researchers via exogenous-editing or recording systems.

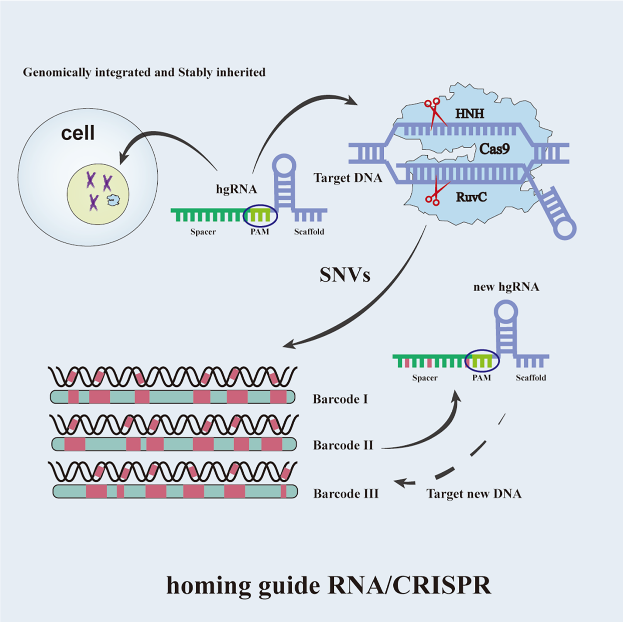

Figure 5. Schematic diagram of DNA lineage barcode generation via continuous hgRNA-CRISPR-Cas9 editing

In hgRNA-based systems, the guide RNA-encoding cassette, which itself contains the PAM, will serve as the target sequence of the hgRNA, allowing Cas9 to edit the genomic locus encoding the guide RNA. As cell divisions proceed, mutations accumulate within the barcode region, creating progressively diversified and heritable records of lineage history.

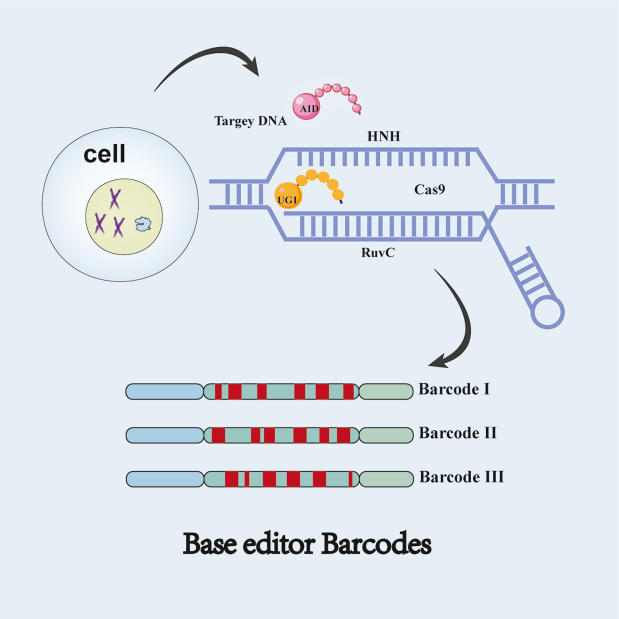

Figure 6. Schematic diagram of lineage barcode generation via SNVs induced by base editors

A base editor is a precise editing tool that fuses a catalytically inactive Cas9 (dCas9) with a deaminase domain, enabling efficient base conversions without introducing DNA double-strand breaks. As illustrated, the editor targets specific genomic sequences (Target DNA), and its deaminase domain directly deaminates bases such as cytosine (C), thereby inducing heritable single-nucleotide variants (SNVs) during DNA replication. These SNVs accumulate in different cells and their progeny, forming unique combinations that serve as high-precision DNA barcodes (as shown in "Barcode I-III") for tracing cell lineage relationships. This method avoids the complex insertions/deletions (indels) associated with double-strand break repair, providing clearer and more easily decodable lineage marks.

Base editor barcode

As illustrated in the Figure 6, single-nucleotide variants (SNVs) generated by base editors can serve as effective barcodes for cell lineage tracing. Base editors, initially designed for precise gene editing based on dCas9, have since been repurposed for lineage tracing. A base editor comprises a DNA-binding domain, a deaminase domain, and a uracil glycosylase inhibitor (UGI) domain. The DNA-binding domain, such as dCas9, targets specific DNA sequences, while the deaminase domain converts adjacent nucleotides, inducing nucleotide changes during DNA replication. The UGI domain inhibits repair mechanisms that might reverse these conversions. Hwang B demonstrated the use of base editors by targeting endogenous L1 (long interspersed nuclear elements) repeat sequences, which are abundant and diverse in the genome, to generate cells with barcode sequences. This experiment confirmed the utility of base editors as tools for cell lineage tracing.[52] Some researchers have further modified base editors to enable selective base conversions across multiple genes or locations in the genome, simplifying multi-site editing and expanding the diversity of generated barcodes.[53] The transformer base editor, for example, can edit DNA in cells with high efficiency, achieving up to 90% editing success in primary human T cells without causing DSBs. This tool has been used to knock out multiple target genes, such as T cell receptor alpha constant (TRAC), CD52, and programmed cell death 1 (PDCD1), with impressive results.[54]

Endogenous natural barcode

Markers derived from endogenous cellular genetic features that can be used to trace clonal or lineage relationships without additional engineering.

mtDNA barcode

As illustrated in the Figure 7, mtDNA is particularly useful for lineage tracing due to its high mutation rate, which is typically 10–100 times higher than nuclear DNA, allowing mtDNA to accumulate mutations during cell proliferation.[55] This makes it an ideal candidate for use as a DNA barcode in cell lineage tracing. Although obtaining the complete mtDNA sequence from a single cell was once a challenge, advancements in single-cell sequencing technology have made it possible to capture mtDNA and detect mutations at single-cell resolution, particularly through techniques like scATAC-seq.[56] Researchers have combined scATAC-seq with scRNA-seq to label individual cells.[42] For example, Leif S. Ludwig and colleagues demonstrated that mtDNA barcodes can accurately identify clonal relationships, using subclones of the hematopoietic TF1 cell line.[41]

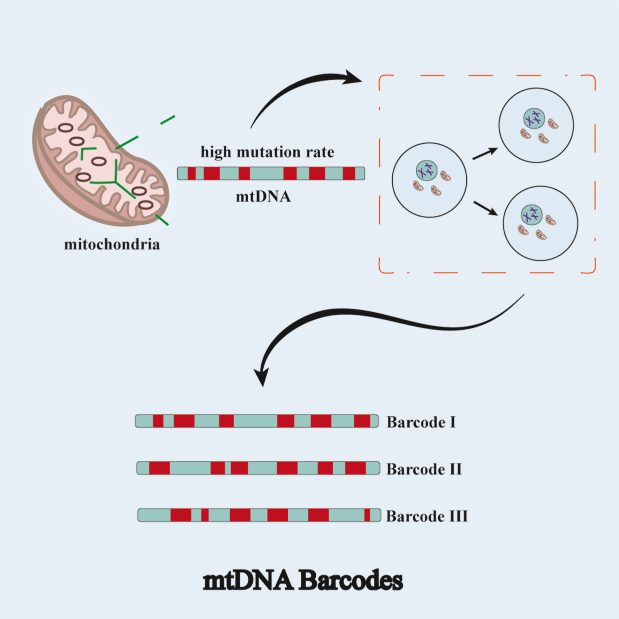

Figure 7. Schematic diagram of utilizing high-frequency mtDNA mutations as natural lineage barcodes

Mitochondrial DNA (mtDNA) serves as an ideal natural tool for lineage tracing due to its high mutation rate (typically 10–100 times higher than nuclear DNA). As illustrated, random point mutations continuously accumulate in mtDNA during cell proliferation. Given the polyploid nature of mtDNA in the cytoplasm and its independent inheritance from the nuclear genome, these mutations can be stably passed on to daughter cells. As lineages diverge, distinct cell branches accumulate unique sets of mutations, forming distinguishable DNA barcodes (as shown in "Barcode I-III"). Serving as intrinsic cellular marks, these barcodes can be used to infer clonal relationships without the need for introducing exogenous genes, making them particularly suitable for short-term, high-resolution lineage tracing studies.

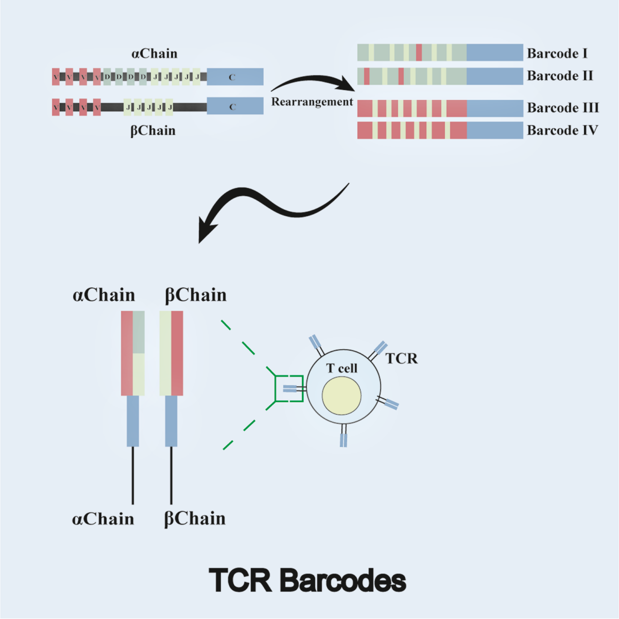

Figure 8. Schematic diagram of TCR gene rearrangement generating natural lineage barcodes

During T cell development, the genes encoding the α and β chains of the T cell receptor (TCR) undergo somatic V(D)J rearrangement to form diverse functional genes. As illustrated, random and irreversible selection and joining occur among the multiple variable (V), diversity (D, primarily in the β chain), and joining (J) gene segments in the germline. Each successful rearrangement event creates a unique and heritable DNA sequence. Consequently, these rearranged TCR sequences serve as natural, high-resolution DNA barcodes (as shown in "Barcode I-IV") for tracking the clonal origin, expansion, and evolutionary dynamics of T cells (e.g., tumor-infiltrating T cells or infused CAR-T cells) at the single-cell level.

TCR barcode

In immune cells, the extensive TCR repertoire, which recognizes both exogenous and endogenous antigens, can serve as a natural DNA barcode for lineage tracing. As illustrated in the Figure 8, due to TCR rearrangement, distinct TCR sequences emerge, providing a means to track T cells over time. Compared to integration barcodes, CARLIN, and SMALT, TCR barcodes are inherently present in αβ T cells because these cells naturally carry TCR clonotypes. This makes TCR barcodes particularly well suited for tracing T cell lineages. Additionally, they can help elucidate T cell clone evolution and identify which clones are associated with therapeutic efficacy. Zhang Lei and colleagues collected T cells from 12 patients with colorectal cancer, obtaining both single-cell transcriptome and TCR-seq data.[57] They used the α and β chains of TCRs as lineage markers, combining this with scRNA-seq to analyze the T cell landscape and differentiation status in patients with colorectal cancer. Similarly, Cao et al. used TCR clonotypes to label the lineage of CAR-T cells, allowing them to track the evolution of CAR-T cells over time in patients with diffuse large B cell lymphoma (DLBCL) undergoing CAR-T therapy.[58] However, due to the high heterogeneity of TCR sequences, most TCRs differ between patients, and shared TCRs are rare. Therefore, TCR barcodes are highly personalized for tracking individual T cell lineages. Moreover, current TCR barcodes can only track T cell clones and cannot trace the complete developmental lineage.

SCLT offers a robust methodological framework for elucidating the interrelationships among clonal origin, cellular states, and fate trajectories within engineered T cell therapies. Presently, the efficacy of engineered T cell therapy hinges not only on the long-term persistence of infused cells *in vivo* but also, critically, on the identification of specific clonal populations capable of sustained expansion and survival. It is essential to discern which cells undergo fate differentiation, experience functional impairment or exhaustion, and which actively contribute to durable anti-tumor responses. By comparing the transcriptional expression profiles of these distinct cellular subsets, it becomes feasible to investigate strategies to mitigate cellular dysfunction or exhaustion, thereby enhancing the participation of engineered T cells in anti-tumor activity. Integrating lineage barcoding with single-cell analysis, SCLT enables the reconstruction of clonal relationships among engineered T cells while concurrently monitoring dynamic alterations in transcriptional states and phenotypic features at single-cell resolution over time. This approach is pivotal for addressing clinical challenges associated with engineered

T cell therapies. For instance, SCLT can elucidate whether long-term resident T cells are sustained by a polyclonal repertoire or dominated by a limited number of clones, and further clarify how distinct clones pursue divergent cellular fates. Additionally, lineage-resolved single-cell analyses facilitate the determination of whether disease relapse and treatment-related toxicities correlate with the evolutionary trajectories of particular clones, rather than reflecting changes at the bulk population level. The utility of SCLT in engineered T cell therapy extends beyond tracking cellular fates to encompass a comprehensive understanding of how individual clones expand, differentiate, adapt, and ultimately impact therapeutic efficacy.

Methods for analyzing single-cell lineage data

Given the vast amount of data generated by SCLT, it is essential to balance algorithm sensitivity with biologically interpretable analysis methods. Commonly used software for analyzing single-cell data includes Seurat[59] and Scanpy,[60] corresponding to the R and Python programming languages, respectively. In many SCLT studies, pseudotime analysis or trajectory inference is further incorporated to relate clonal relationships to dynamic changes in cell state and fate. Trajectory analysis is a key technique for understanding cell dynamics and is a central approach in single-cell research of cell developmental trajectories. To date, more than 70 trajectory inference methods have been developed for single-cell data analysis.[61] Benchmark studies have shown that methods, such as Slingshot,[62] TSCAN,[63] and Monocle DDRTree[64] perform favorably across multiple evaluation settings, particularly in terms of accuracy and scalability, although no single method is uniformly optimal for all datasets. As shown in Table S2, these trajectory inference methods provide complementary strategies for reconstructing dynamic cell-state transitions from single-cell transcriptomic data. Although these methods do not directly infer clonal lineages, they are often combined with lineage barcode information, thereby linking transcriptional state transitions to clonal relationships. These methods offer more reliable inference results when dealing with both linear and nonlinear trajectories.[61] As single-cell technologies evolve, numerous data analysis tools have emerged, each with distinct features and advantages. Selecting the appropriate methods based on the specific characteristics of the data, along with using multiple methods for validation, enhances the reliability and accuracy of conclusions. These methods infer trajectories based on the static gene expression states of single cells, thereby reconstructing the fate trajectories of cells along continuous paths. However, this does not equate to lineage tracing; true lineage tracing requires determining cell lineage through lineage barcodes. In SCLT, trajectory inference integrates authentic lineage information within a dynamic framework of cell state changes. This approach facilitates the identification of fate boundaries and early markers.

Beyond conventional trajectory inference, several lineage-aware computational frameworks have been developed to integrate barcoding information with transcriptomic profiles, thereby improving the reconstruction of clonal relationships and developmental trajectories.

Comparative utility of lineage-tracing strategies in engineered T cells

As illustrated in Table S3, CRISPR-Cas9-based dynamic barcode systems—such as LINNAEUS, CARLIN, DARLIN, and hgRNA—are categorized under the exogenous barcode strategy. These systems generate prospective and controllable lineage barcodes by continuously accumulating mutations at predetermined loci through inducible CRISPR-mediated editing. In 2018, Spanjaard et al. employed transgenic reporter target sites in zebrafish, introducing Cas9-induced scars during early developmental stages.[46] These scars were subsequently analyzed via single-cell RNA sequencing (scRNA-seq) to correlate cell types with their developmental origins. Building on the LINNAEUS framework, Bowling et al. (2020) developed CARLIN, a system designed to enhance inducibility, generalizability, and enable *in vivo* lineage tracing in murine models.[47] More recently, in 2023, DARLIN was introduced as an advancement of CARLIN, offering increased barcode capacity and improved barcode generation capabilities.[65] Despite these advancements, the practical application of CRISPR barcodes in engineered T cells remains constrained by several challenges, including genotoxicity associated with editing, off-target effects, barcode stability, and the potential influence of genome engineering on T cell functionality, proliferative capacity, and therapeutic efficacy. These issues are particularly pertinent for CAR-T and TCR-T therapies, which necessitate complex *ex vivo* manipulations and sustained persistence following reinfusion.

LINNAEUS

Similar to the SMALT system, the generation of scar barcodes depends on the cell state and promoter strength and is not directly related to mitosis, so the reconstructed lineage trees rarely capture all cell division events. Additionally, scar barcode generation does not continue indefinitely and stops early, which leads to incomplete coverage of the terminal branches of the lineage. Some researchers have pointed out that introducing "barcode + UMI + scar sequence" to correct dropout can improve the reliability of barcode and lineage tree construction.[66] At the same time, methods like moslin more fully integrate lineage constraints into cross-time point state alignment and trajectory inference, enhancing the interpretability of "state–fate" relationships.[67]

CARLIN/DARLIN

Essentially, CARLIN is a CRISPR indel barcode, so it faces the same issues as LINNAEUS (such as indel bias, homoplasy, asynchronous editing rates relative to cell division, dropout, and single-cell recovery rates).[66] Building on CARLIN's Cas9 dynamic barcode, DARLIN introduces TDT (terminal deoxynucleotidyl transferase) and expands the target site array to 30 sites, greatly increasing barcode diversity.[65] This approach can improve the issue of barcode homoplasy, where different clonal cells share the same barcode. DARLIN can also perform multi-omics analysis. Camellia-seq is a four-modality single-cell joint profiling method that can simultaneously obtain lineage barcodes, gene expression, chromatin accessibility, and DNA methylation from the same single cell. Some researchers separate cells into cytoplasm and nucleus for sequencing: the cytoplasmic

fraction is used for transcriptome and lineage barcode analysis, while the nuclear fraction undergoes single-cell bisulfite sequencing after GpC methyltransferase labeling. This allows simultaneous profiling of open chromatin, CpG methylation, and RNA,[65] but it requires deep sequencing coverage and is costly.

hgRNA barcode

Unlike LINNAEUS and CARLIN, hgRNA/CRISPR does not require the prior insertion of a reporter gene; the homing CRISPR barcode accumulates indels through the self-updating of the gRNA. However, the self-targeting nature of the gRNA can lead to barcode loss. Additionally, random integration of the hgRNA cassette into the genome may disrupt or affect the expression of nearby genes. Theresa B. Loveless and colleagues proposed a method called CHYRON.[68] They added TDT to the hgRNA system, enabling random addition of new nucleotides at DNA break ends. The PAM site of Cas9 in the hgRNA locus is fixed, so after each self-targeting event, the new insertions sequentially stack downstream of the previous insertion. In other words, CHYRON records ordered insertions rather than random indels. This reduces the likelihood that indels in the hgRNA will target essential genes. However, currently CHYRON mainly relies on DNA-level sequencing readout and cannot be integrated with scRNA-seq. Excessively long gRNAs can reduce the cleavage efficiency of Cas proteins.[69] Mirazul Islam and colleagues transformed hgRNA into a target that can enter single-cell analysis together with the transcriptome and addressed the issue of sparse single-cell data for hgRNA.[70] At present, hgRNA is mainly used for studying disease mechanisms, developmental biology, and preclinical model validation and is not yet suitable as a clinical barcode system directly applicable to patients. In 2024, hgRNA barcodes were first incorporated into single-cell platforms, enabling the concurrent inference of cellular states, clonal lineage relationships, and temporal sequencing of events *in vivo*.[70] This development underscores the significant potential of hgRNA barcodes, although their technology remains in an emergent stage and has yet to reach full maturity.

The fundamental principle underlying substitution mutation barcodes (such as SMALT and Cas9-deaminase) involves the accumulation of lineage information via base substitutions. In comparison to conventional indel recorders, this approach offers a significant advantage by minimizing the occurrence of large fragment deletions that can result in the loss of lineage data, thereby facilitating more reliable long-term recording.

SMALT

Because substitution patterns resemble SNP data, SMALT-derived barcodes can be analyzed using established phylogenetic frameworks (e.g., maximum likelihood inference and bootstrap support).[49,71] Nevertheless, SMALT is not uniformly random: AID exhibits strong sequence-context preferences, making certain cytosines more mutable than others.[72] Such context bias increases the risk of convergent edits and barcode homoplasy, particularly at large scale, potentially confounding lineage reconstruction if not properly modeled.[50,72] In addition, the effective writing rate is not an intrinsic clock; it is influenced by AID dosage/expression, targeting efficiency, and the balance of uracil-processing repair pathways, such that recording density may correlate with proliferative history but can vary across cell states and contexts.[73] Due to the progressive accumulation of these mutation types during DNA replication, they are especially well suited for tracing the history of cellular division and clonal expansion. This approach enables the investigation of questions such as whether, following reinfusion, CAR-T cell expansion is predominantly driven by a limited number of dominant clones or sustained collectively by multiple clones, as well as identifying which initial cells contribute to long-term persistence. Such applications represent a logical extension of SMALT's capacity for high-resolution lineage reconstruction.

Base editor barcode

The base editor barcode exhibits similarities to SMALT in that it does not depend on DSBs but instead produces barcodes via the substitution mutation mechanism mediated by nCas9/deaminase. In contrast to the prevalent large fragment deletions, site collapses, and sequence dropouts commonly observed in CRISPR-based barcodes, the base editor barcode continuously introduces substitution mutations within the original barcode sequence. Nevertheless, it shares a limitation with SMALT wherein the rate of barcode generation is not synchronized with cellular mitosis. Although the base editor barcode circumvents extensive deletions caused by indels, its mutational pattern is neither entirely uniform nor random; it is influenced by the distribution of editable motifs, the sequence context preferences of the deaminase enzyme, and the constraints imposed by the editing window. Currently, the base editor barcode technology remains in a developmental stage; effective application at the single-cell level necessitates sufficiently deep sequencing coverage and carefully optimized barcode design.

Integration barcode

Compared to SMALT, LINNAEUS, and Polylox, integration barcodes are simpler to construct in large libraries and can label a large number of cells at once, making them suitable for immune cell expansion and fate analysis. Some researchers have proposed a method that combines integration barcodes with single-cell transcriptomics—LARRY (lineage and RNA recovery)—and applied this method to mouse hematopoietic cell differentiation, visually demonstrating the connection between the scRNA-seq state map and the cell fate map.[40,56] Taha B. Hayal and colleagues incorporated a highly complex genetic barcode library into a CAR lentiviral vector, prepared barcode-labeled CAR-NK cells *in vitro*, and used them to track CAR-NK cells in NSG mice.[74] Several studies have conducted TCR beta-chain sequencing (TCRβ-seq), integration site analysis, and scRNA-seq on infusion products (IP) as well as on CD8 CAR-T cells present in the peripheral blood of patients undergoing CD19 CAR-T immunotherapy. These investigations focus on examining the clonal diversity and clonal dynamics of CAR-T

cells both prior to and following reinfusion.[75] Considering safety and stability, natural barcodes are currently suitable for lineage tracing in human clinical settings. Integration barcodes are more commonly used in research models, cell product evaluation, and preclinical validation. The integration barcode fundamentally arises from the stochastic incorporation of viral integration sites or artificially engineered barcode cassettes delivered by integrative vectors. Such random integration events have the potential to influence host gene regulation and cellular adaptability. Consequently, a thorough assessment of the safety associated with these insertion sites is imperative.

Polylox barcode

Relative to alternative strategy types, the Polylox barcode system enables precise regulation of barcode generation within living organisms. For instance, Allen M.E. et al. developed a light-activated and drug-activated CAR system based on the Cre-loxP method (TamPA-Cre system), using tamoxifen and blue light to activate Cre recombinase, thus driving CAR expression.[76] The essence of the Polylox barcode is DNA recombination; it does not depend on cellular transcriptional expression and is suitable for long-term *in vivo* tracking. Unlike traditional Cre fate mapping, Polylox can achieve high-resolution endogenous barcode labeling *in vivo* and has been applied to HSC fate mapping.[77] The diversity of the Polylox barcode comes from the recombination of preset *loxP* segments, so the barcode diversity is determined by the cassette structure. Some researchers have improved Polylox by transforming the original Polylox barcode, which could only be read at the DNA level, into the PolyloxExpress system that can simultaneously read both the 'barcode + transcriptome' in scRNA-seq, thereby obtaining transcriptome information and lineage tracing within the same single HSC cell.[78] From the standpoint of engineered T cells, the current application of this methodology is primarily limited to conditional gene switches or circuit control, as well as pre-constructed models in transgenic mice, and it has not yet been directly implemented in clinical settings. Consequently, it does not represent a widely adopted strategy for SCLT. Furthermore, its practical utility within the T cell system is demonstrably inferior to approaches based on TCR analysis, genomic integration, or mtDNA profiling. Therefore, this strategy remains a non-mainstream approach at present and requires further refinement and development.

mtDNA barcode

The mtDNA barcode represents an intrinsic endogenous marker that obviates the need for the exogenous labeling of cells. This approach leverages the somatic mutations that naturally accrue in mtDNA throughout physiological and pathological processes to infer lineage relationships and clonal origins. Unlike CRISPR-based barcodes, mtDNA barcodes do not require the introduction of exogenous genes, but they are limited by mitochondrial inheritance patterns and tissue applicability, making them more suitable for short-term tracking studies of primary cells. As illustrated in the Table S3, recent advancements have extended mtDNA lineage tracing to include frozen samples and spatial transcriptomics techniques,[79] thereby facilitating the concurrent analysis of spatial localization, transcriptional states, and clonal relationships within intact human tissues. This development enhances the applicability of mtDNA barcodes in clinical cohorts and pathological specimens, surpassing the utility of most prospective engineered barcodes. Although the feasibility of this approach has been demonstrated in T cells, it has yet to become a widely adopted lineage tracing method within the CAR-T cell research domain.[41] Nonetheless, mtDNA barcoding currently faces notable limitations. Primarily, its detection remains dependent on sequencing depth and the specific sequencing platform employed. Additionally, emerging evidence suggests that mtDNA variations at the single-cell level may not represent entirely neutral genetic mutations across diverse environmental contexts.

TCR barcode

TCR barcodes and mtDNA barcodes are both classified as endogenous barcodes; however, unlike mtDNA barcodes, TCR barcodes represent clonotype identities following V(D)J recombination in mature T cells, a process intimately linked to antigen specificity. Consequently, TCR barcodes are particularly well suited for investigations of engineered T cell lineages. The STARTRAC methodology has demonstrated that integrating scRNA-seq with TCR-seq enables effective characterization of clonal expansion among tumor-infiltrating $CD4^+$ and $CD8^+$ T cells. In the context of CAR-T cells, TCR barcodes have been employed to analyze clonal dynamics within both CAR-T and non-CAR-T cell populations in patient samples, thereby enhancing their applicability in clinical settings relative to most exogenous barcoding approaches. Nonetheless, additional molecular recorders are required to further delineate hierarchical differentiation within individual clonotypes, as TCR barcodes are limited to tracking clonal hierarchies subsequent to TCR rearrangement.

Regarding the temporal scope of lineage tracing, cumulative barcodes are generally more appropriate for long-term tracking in preclinical investigations, as these studies permit engineered labeling and necessitate high-resolution lineage reconstruction. Conversely, endogenous barcodes—particularly mtDNA variants and TCR clonotypes—are typically more practical for patient-derived samples and clinically oriented research due to their acquisition requiring fewer manipulations and involving reduced regulatory complexity. For expanded, ongoing, or short-term monitoring of tumor infiltration, TCR and mtDNA barcodes can already yield valuable insights, whereas long-term reconstruction of branching clonal histories usually benefits from specialized synthetic or evolving barcode systems. Nonetheless, in scenarios where endogenous TCR loci are disrupted or replaced, such as in certain allogeneic or extensively engineered T cell products, the effectiveness of TCR-based lineage tracing may be compromised.

Practical implications of SCLT for engineered T cell immunotherapy

From a practical standpoint, the utility of SCLT in engineered T cell therapy resides in its capacity to correlate clonal history with

therapeutic outcomes. In preclinical investigations, lineage tracing enables the identification of input clones that preferentially expand, persist, infiltrate tumors, or transition into dysfunctional states during CAR-T or TCR-T treatments. During the cell manufacturing process, SCLT facilitates the detection of clonal bottlenecks, disproportionate expansion of minor progenitor subpopulations, and lineage biases introduced by activation, transduction, and *in vitro* culture conditions. At the translational research level, integrating lineage data with single-cell transcriptomic profiles assists in differentiating durable and efficacious therapeutic clones from transient or exhausted cell populations, thereby enhancing the understanding of relapse mechanisms, treatment failure, and inter-patient variability. More broadly, SCLT contributes to the rational optimization of engineered T cell products by elucidating clonal evolutionary trajectories associated with therapeutic efficacy, persistence, or toxicity.

While lineage tracing is not inherently a direct safety control mechanism, it serves a critical role in elucidating whether adverse toxic responses or therapeutic failures correlate with particular patterns of clonal expansion, differentiation pathways, or persistence states within engineered T cell populations.

DISCUSSION

The primary rationale for the enhancement of immunotherapy through SCLT in clinical settings lies in its ability to translate observations of efficacy, relapse, and toxicity from the population level to the resolution of specific cellular clones, distinct gene expression states, and defined cellular fate trajectories. In 2020, Alyssa Sheih and colleagues performed an integrative analysis combining TCRB clone dynamics,[75] integration site mapping, and scRNA-seq of CD19 CAR-T cells. Their findings revealed that the CAR-T cell clones exhibiting significant expansion post-infusion predominantly originated from cell populations within the infusion product characterized by elevated expression of genes associated with cytotoxicity and proliferation. This evidence suggests that SCLT can discern whether the engineered T cell infusion product is composed of a polyclonal repertoire or dominated by a limited number of clones. Furthermore, by identifying more favorable cellular subpopulations through detailed single-cell clone and state profiling, SCLT offers valuable insights for optimizing therapeutic products and improving clinical efficacy. Additionally, the technique facilitates the evaluation of engineered T cell product effectiveness by quantifying the relative abundance of specific cell subpopulations within the infusion material. In 2023, Nathaniel D. Anderson and colleagues conducted single-cell gene expression profiling and TCR-seq on CD19 CAR-T cell infusion products,[80] as well as on blood and bone marrow samples obtained five years post-infusion. Their investigation revealed that CAR-T cells expressing a distinct set of identical genes were present in all patients who attained complete remission (CR). Furthermore, they identified that these CAR-T cells emerged through convergent evolution following the long-term development of multiple diverse clones. These findings suggest that SCLT is a valuable tool for elucidating the *in vivo* cellular fate and adverse effects of engineered T cells, enhancing the understanding of resistance mechanisms in patients experiencing cancer relapse, and informing future research directions in the field of engineered T cell therapies.[81–83]

Building upon this foundation, a promising avenue for future research involves the enhanced integration of targeted gene editing—commonly employed in engineered T cell therapies—with SCLT methodologies. For instance, in engineered T cell platforms such as TCR-T therapies or those necessitating the elimination of endogenous TCR interference, CRISPR-mediated editing at loci including TRAC and T cell receptor beta constant (TRBC) not only serves to optimize the expression of therapeutic receptors but also has the potential to generate traceable genetic markers via the induced insertions and deletions (indels).[84–86] When combined with scRNA-seq and TCR-seq, this strategy could enable concurrent analysis of the interplay between engineered receptor design, endogenous TCR modifications, and clonal fate dynamics within a unified analytical framework. Nonetheless, the implementation of this approach necessitates rigorous validation to assess the diversity of indels at the TRAC and TRBC-editing sites, the stability of these genetic barcodes, and their capacity to facilitate high-resolution lineage reconstruction.

Looking forward, the continued development of SCLT in engineered T cells will likely rely on molecular readout strategies with higher structural resolution. Relative to short-read approaches, third-generation long-read sequencing may enable more complete characterization of complex barcode architectures, integration-site structures, edited allelic configurations at loci such as TRAC and TRBC, and full-length TCR or CAR transcripts, thereby reducing barcode ambiguity and enhancing clonal reconstruction.[87–89] In studies that aim to jointly resolve lineage relationships, receptor structure, editing outcomes, and transcriptional state, such platforms may serve as an important technological bridge between mechanistic studies and translational applications. However, their broader implementation will require further improvements in cost, throughput, sequencing accuracy, and compatibility with single-cell multi-omics frameworks.

In addition, translational application of lineage-tracing strategies in engineered T cell therapies must be considered in light of manufacturing and regulatory constraints. Introducing exogenous barcoding constructs into therapeutic T cells may increase product complexity, necessitate additional quality-control procedures, and raise biosafety and regulatory concerns related to vector copy number, genomic integration sites, and product consistency. These issues are particularly relevant in the manufacture of clinical-grade CAR-T and TCR-T products, where unnecessary genetic manipulation is generally best avoided. In this context, endogenous lineage markers, such as mtDNA variants or TCR clonotypes, may provide a lower-burden alternative for correlative studies in patient-derived samples, although they typically offer less experimental control than dedicated synthetic barcoding systems. Future progress will likely depend on how effectively

lineage resolution can be balanced against manufacturability, safety, and clinical feasibility.

Overall, the field is shifting from proof of concept lineage-recording studies toward question-driven applications in engineered T cell immunotherapy. The next phase of development will require not only higher barcode resolution and deeper multi-omic integration but also greater attention to manufacturability, regulatory acceptability, and clinical interpretability. In this setting, highly engineered synthetic barcodes and minimally perturbative endogenous barcodes should not be viewed as competing solutions, but rather as complementary tools suited to different stages of CAR-T and TCR-T research and translation.

ACKNOWLEDGMENTS

This work was supported by Natural Science Foundation of Guangdong Province (2024A1515011201), Innovation Team project in regular colleges and universities of Guangdong Province (natural science) (2023KCXTD021).

AUTHOR CONTRIBUTIONS

H.S., W.Z., S.L., and J.W. conceived and designed the structure of this review; J.W., Z.L., J.C., H.L., F.C., L.T., and X.C. drafted the article; the table was organized by J.W. and Z.L.; the figure was drawn by J.C., H.L., and F.C.; H.S. and W.Z. reviewed and edited the article and supervised the study. All authors reviewed and approved the final version of this article; H.S., S.L., and W.Z. are co-corresponding authors.

DECLARATION OF INTERESTS

The authors declare that the research was conducted in the absence of any commercial or financial relationships that could be construed as a potential conflict of interest.

SUPPLEMENTAL INFORMATION

Supplemental information can be found online at https://doi.org/10.1016/j.omta.2026.201802.

REFERENCES

1. Deeks, S.G., Wagner, B., Anton, P.A., Mitsuyasu, R.T., Scadden, D.T., Huang, C., Macken, C., Richman, D.D., Christopherson, C., June, C.H., et al. (2002). A phase II randomized study of HIV-specific T-cell gene therapy in subjects with undetectable plasma viremia on combination antiretroviral therapy. Mol. Ther. *5*, 788–797. https://doi.org/10.1006/mthe.2002.0611.

2. Kershaw, M.H., Westwood, J.A., Parker, L.L., Wang, G., Eshhar, Z., Mavroukakis, S.A., White, D.E., Wunderlich, J.R., Canevari, S., Rogers-Freezer, L., et al. (2006). A phase I study on adoptive immunotherapy using gene-modified T cells for ovarian cancer. Clin. Cancer Res. *12*, 6106–6115. https://doi.org/10.1158/1078-0432.Ccr-06-1183.

3. Porter, D.L., Levine, B.L., Kalos, M., Bagg, A., and June, C.H. (2011). Chimeric antigen receptor-modified T cells in chronic lymphoid leukemia. N. Engl. J. Med. *365*, 725–733. https://doi.org/10.1056/NEJMoa1103849.

4. FDA. Approved Cellular and Gene Therapy Products. https://www.fda.gov/vaccines-blood-biologics/cellular-gene-therapy-products/approved-cellular-and-gene-therapy-products?utm_source=copilot.com.

5. EMA (2018). First two CAR-T cell medicines recommended for approval in the European Union. https://www.ema.europa.eu/en/news/first-two-car-t-cell-medicines-recommended-approval-european-union.

6. EMA (2022). New gene therapy treatment for patients with relapsed or refractory large B-cell lymphoma. https://www.ema.europa.eu/en/news/new-gene-therapy-treatment-patients-relapsed-or-refractory-large-b-cell-lymphoma.

7. (2022). New gene therapy to treat adult patients with multiple myeloma. https://www.ema.europa.eu/en/news/new-gene-therapy-treat-adult-patients-multiple-myeloma,E.

8. NMPA (2023). 2023年度药品审评报告. https://www.nmpa.gov.cn/directory/web/nmpa/images/1707039824076019627.pdf.

9. Atsavapranee, E.S., Billingsley, M.M., and Mitchell, M.J. (2021). Delivery technologies for T cell gene editing: Applications in cancer immunotherapy. EBioMedicine *67*, 103354. https://doi.org/10.1016/j.ebiom.2021.103354.

10. Zhang, H., Li, X., Yang, G., Ru, J., and Liu, T. (2018). Emodin protects hyperglycemia-induced injury in PC-12 cells by up-regulation of miR-9. Mol. Cell. Endocrinol. *474*, 194–200. https://doi.org/10.1016/j.mce.2018.03.009.

11. Feins, S., Kong, W., Williams, E.F., Milone, M.C., and Fraietta, J.A. (2019). An introduction to chimeric antigen receptor (CAR) T-cell immunotherapy for human cancer. Am. J. Hematol. *94*, S3–S9. https://doi.org/10.1002/ajh.25418.

12. Zhang, Y., Zhang, Z., Ding, Y., Fang, Y., Wang, P., Chu, W., Jin, Z., Yang, X., Wang, J., Lou, J., and Qian, Q. (2021). Phase I clinical trial of EGFR-specific CAR-T cells generated by the piggyBac transposon system in advanced relapsed/refractory non-small cell lung cancer patients. J. Cancer Res. Clin. Oncol. *147*, 3725–3734. https://doi.org/10.1007/s00432-021-03613-7.

13. Martinez, M., and Moon, E.K. (2019). CAR T Cells for Solid Tumors: New Strategies for Finding, Infiltrating, and Surviving in the Tumor Microenvironment. Front. Immunol. *10*, 128. https://doi.org/10.3389/fimmu.2019.00128.

14. Livet, J., Weissman, T.A., Kang, H., Draft, R.W., Lu, J., Bennis, R.A., Sanes, J.R., and Lichtman, J.W. (2007). Transgenic strategies for combinatorial expression of fluorescent proteins in the nervous system. Nature *450*, 56–62. https://doi.org/10.1038/nature06293.

15. Porter, S.N., Baker, L.C., Mittelman, D., and Porteus, M.H. (2014). Lentiviral and targeted cellular barcoding reveals ongoing clonal dynamics of cell lines in vitro and in vivo. Genome Biol. *15*, R75. https://doi.org/10.1186/gb-2014-15-5-r75.

16. Conklin, E.G. (1897). THE EMBRYOLOGY OF CREPIDULA, A CONTRIBUTION TO THE CELL LINEAGE AND EARLY DEVELOPMENT OF SOME MARINE GASTEROPODS. J. Morphol. *13*, 1–226.

17. Lesseps, R.J., van Kessel, A.H.M.G., and Denucé, J.M. (1975). Cell patterns and cell movements during early development of an annual fish, Nothobranchius neumanni. J. Exp. Zool. *193*, 137–146. https://doi.org/10.1002/jez.1401930203.

18. Steinhauser, M.L., and Lechene, C.P. (2013). Quantitative imaging of subcellular metabolism with stable isotopes and multi-isotope imaging mass spectrometry. Semin. Cell Dev. Biol. *24*, 661–667. https://doi.org/10.1016/j.semcdb.2013.05.001.

19. Gimlich, R.L., and Cooke, J. (1983). Cell lineage and the induction of second nervous systems in amphibian development. Nature *306*, 471–473. https://doi.org/10.1038/306471a0.

20. Slack, J.M.W., Dale, L., and Smith, J.C. (1984). Analysis of embryonic induction by using cell lineage markers. Philos. Trans. R. Soc. Lond. B Biol. Sci. *307*, 331–336. https://doi.org/10.1098/rstb.1984.0135.

21. Weisblat, D.A., Sawyer, R.T., and Stent, G.S. (1978). Cell lineage analysis by intracellular injection of a tracer enzyme. Science *202*, 1295–1298. https://doi.org/10.1126/science.725606.

22. Jacobson, M., and Hirose, G. (1978). Origin of the retina from both sides of the embryonic brain: a contribution to the problem of crossing at the optic chiasma. Science *202*, 637–639. https://doi.org/10.1126/science.705349.

23. Hsu, Y.C. (2015). Theory and Practice of Lineage Tracing. Stem Cell. *33*, 3197–3204. https://doi.org/10.1002/stem.2123.

24. Demeulemeester, J., De Rijck, J., Gijsbers, R., and Debyser, Z. (2015). Retroviral integration: Site matters: Mechanisms and consequences of retroviral integration site selection. Bioessays *37*, 1202–1214. https://doi.org/10.1002/bies.201500051.

25. Biddy, B.A., Kong, W., Kamimoto, K., Guo, C., Waye, S.E., Sun, T., and Morris, S.A. (2018). Single-cell mapping of lineage and identity in direct reprogramming. Nature *564*, 219–224. https://doi.org/10.1038/s41586-018-0744-4.

26. Sun, J., Ramos, A., Chapman, B., Johnnidis, J.B., Le, L., Ho, Y.J., Klein, A., Hofmann, O., and Camargo, F.D. (2014). Clonal dynamics of native haematopoiesis. Nature *514*, 322–327. https://doi.org/10.1038/nature13824.

27. Tang, F., Barbacioru, C., Wang, Y., Nordman, E., Lee, C., Xu, N., Wang, X., Bodeau, J., Tuch, B.B., Siddiqui, A., et al. (2009). mRNA-Seq whole-transcriptome analysis of a single cell. Nat. Methods *6*, 377–382. https://doi.org/10.1038/nmeth.1315.

28. Navin, N., Kendall, J., Troge, J., Andrews, P., Rodgers, L., McIndoo, J., Cook, K., Stepansky, A., Levy, D., Esposito, D., et al. (2011). Tumour evolution inferred by single-cell sequencing. Nature *472*, 90–94. https://doi.org/10.1038/nature09807.

29. Stoeckius, M., Hafemeister, C., Stephenson, W., Houck-Loomis, B., Chattopadhyay, P.K., Swerdlow, H., Satija, R., and Smibert, P. (2017). Simultaneous epitope and transcriptome measurement in single cells. Nat. Methods *14*, 865–868. https://doi.org/10.1038/nmeth.4380.

30. Hasin, Y., Seldin, M., and Lusis, A. (2017). Multi-omics approaches to disease. Genome Biol. *18*, 83. https://doi.org/10.1186/s13059-017-1215-1.

31. Azizi, E., Carr, A.J., Plitas, G., Cornish, A.E., Konopacki, C., Prabhakaran, S., Nainys, J., Wu, K., Kiseliovas, V., Setty, M., et al. (2018). Single-Cell Map of Diverse Immune Phenotypes in the Breast Tumor Microenvironment. Cell *174*, 1293–1308.e36. https://doi.org/10.1016/j.cell.2018.05.060.

32. Lavin, Y., Kobayashi, S., Leader, A., Amir, E.a.D., Elefant, N., Bigenwald, C., Remark, R., Sweeney, R., Becker, C.D., Levine, J.H., et al. (2017). Innate Immune Landscape in Early Lung Adenocarcinoma by Paired Single-Cell Analyses. Cell *169*, 750–765.e17. https://doi.org/10.1016/j.cell.2017.04.014.

33. Poznanski, S.M., Singh, K., Ritchie, T.M., Aguiar, J.A., Fan, I.Y., Portillo, A.L., Rojas, E.A., Vahedi, F., El-Sayes, A., Xing, S., et al. (2021). Metabolic flexibility determines human NK cell functional fate in the tumor microenvironment. Cell Metab. *33*, 1205–1220.e5. https://doi.org/10.1016/j.cmet.2021.03.023.

34. Kim, M.C., Borcherding, N., Ahmed, K.K., Voigt, A.P., Vishwakarma, A., Kolb, R., Kluz, P.N., Pandey, G., De, U., Drashansky, T., et al. (2021). CD177 modulates the function and homeostasis of tumor-infiltrating regulatory T cells. Nat. Commun. *12*, 5764. https://doi.org/10.1038/s41467-021-26091-4.

35. Liu, B., Hu, X., Feng, K., Gao, R., Xue, Z., Zhang, S., Zhang, Y., Corse, E., Hu, Y., Han, W., and Zhang, Z. (2022). Temporal single-cell tracing reveals clonal revival and expansion of precursor exhausted T cells during anti-PD-1 therapy in lung cancer. Nat. Cancer *3*, 108–121. https://doi.org/10.1038/s43018-021-00292-8.

36. Zhang, H., Li, Y., Ibáñez, C.F., and Xie, M. (2024). Perirenal adipose tissue contains a subpopulation of cold-inducible adipocytes derived from brown-to-white conversion. eLife *13*, RP93151. https://doi.org/10.7554/eLife.93151.

37. Gaublomme, J.T., Li, B., McCabe, C., Knecht, A., Yang, Y., Drokhlyansky, E., Van Wittenberghe, N., Waldman, J., Dionne, D., Nguyen, L., et al. (2020). Author Correction: Nuclei multiplexing with barcoded antibodies for single-nucleus genomics. Nat. Commun. *11*, 5619. https://doi.org/10.1038/s41467-020-19357-w.

38. Buenrostro, J.D., Wu, B., Litzenburger, U.M., Ruff, D., Gonzales, M.L., Snyder, M.P., Chang, H.Y., and Greenleaf, W.J. (2015). Single-cell chromatin accessibility reveals principles of regulatory variation. Nature *523*, 486–490. https://doi.org/10.1038/nature14590.

39. Wang, W., Fasolino, M., Cattau, B., Goldman, N., Kong, W., Frederick, M.A., McCright, S.J., Kiani, K., Fraietta, J.A., and Vahedi, G. (2020). Joint profiling of chromatin accessibility and CAR-T integration site analysis at population and single-cell levels. Proc. Natl. Acad. Sci. USA *117*, 5442–5452. https://doi.org/10.1073/pnas.1919259117.

40. Weinreb, C., Rodriguez-Fraticelli, A., Camargo, F.D., and Klein, A.M. (2020). Lineage tracing on transcriptional landscapes links state to fate during differentiation. Science *367*, eaaw3381. https://doi.org/10.1126/science.aaw3381.

41. Ludwig, L.S., Lareau, C.A., Ulirsch, J.C., Christian, E., Muus, C., Li, L.H., Pelka, K., Ge, W., Oren, Y., Brack, A., et al. (2019). Lineage Tracing in Humans Enabled by Mitochondrial Mutations and Single-Cell Genomics. Cell *176*, 1325–1339.e22. https://doi.org/10.1016/j.cell.2019.01.022.

42. Lareau, C.A., Ludwig, L.S., Muus, C., Gohil, S.H., Zhao, T., Chiang, Z., Pelka, K., Verboon, J.M., Luo, W., Christian, E., et al. (2021). Massively parallel single-cell mitochondrial DNA genotyping and chromatin profiling. Nat. Biotechnol. *39*, 451–461. https://doi.org/10.1038/s41587-020-0645-6.

43. Pai, J.A., Hellmann, M.D., Sauter, J.L., Mattar, M., Rizvi, H., Woo, H.J., Shah, N., Nguyen, E.M., Uddin, F.Z., Quintanal-Villalonga, A., et al. (2023). Lineage tracing reveals clonal progenitors and long-term persistence of tumor-specific T cells during immune checkpoint blockade. Cancer Cell *41*, 776–790.e7. https://doi.org/10.1016/j.ccell.2023.03.009.

44. Orban, P.C., Chui, D., and Marth, J.D. (1992). Tissue- and site-specific DNA recombination in transgenic mice. Proc. Natl. Acad. Sci. USA *89*, 6861–6865. https://doi.org/10.1073/pnas.89.15.6861.

45. Schmidt, M., Hoffmann, G., Wissler, M., Lemke, N., Müssig, A., Glimm, H., Williams, D.A., Ragg, S., Hesemann, C.U., and von Kalle, C. (2001). Detection and direct genomic sequencing of multiple rare unknown flanking DNA in highly complex samples. Hum. Gene Ther. *12*, 743–749. https://doi.org/10.1089/104303401750148649.

46. Spanjaard, B., Hu, B., Mitic, N., Olivares-Chauvet, P., Janjuha, S., Ninov, N., and Junker, J.P. (2018). Simultaneous lineage tracing and cell-type identification using CRISPR-Cas9-induced genetic scars. Nat. Biotechnol. *36*, 469–473. https://doi.org/10.1038/nbt.4124.

47. Bowling, S., Sritharan, D., Osorio, F.G., Nguyen, M., Cheung, P., Rodriguez-Fraticelli, A., Patel, S., Yuan, W.C., Fujiwara, Y., Li, B.E., et al. (2020). An Engineered CRISPR-Cas9 Mouse Line for Simultaneous Readout of Lineage Histories and Gene Expression Profiles in Single Cells. Cell *181*, 1410–1422.e27. https://doi.org/10.1016/j.cell.2020.04.048.

48. Abdullah, L., Emiliani, F.E., Vaidya, C.M., Stuart, H., Musial, S.C., Kolling, F.W., Obar, J.J., Rosato, P.C., Ackerman, M.E., Song, L., et al. (2025). The endogenous antigen-specific CD8(+) T cell repertoire is composed of unbiased and biased clonotypes with differential fate commitments. Immunity *58*, 601–615.e9. https://doi.org/10.1016/j.immuni.2025.02.001.

49. Liu, K., Deng, S., Ye, C., Yao, Z., Wang, J., Gong, H., Liu, L., and He, X. (2021). Mapping single-cell-resolution cell phylogeny reveals cell population dynamics during organ development. Nat. Methods *18*, 1506–1514. https://doi.org/10.1038/s41592-021-01325-x.

50. Chen, C., Liao, Y., and Peng, G. (2022). Connecting past and present: single-cell lineage tracing. Protein Cell *13*, 790–807. https://doi.org/10.1007/s13238-022-00913-7.

51. Kalhor, R., Mali, P., and Church, G.M. (2017). Rapidly evolving homing CRISPR barcodes. Nat. Methods *14*, 195–200. https://doi.org/10.1038/nmeth.4108.

52. Hwang, B., Lee, W., Yum, S.Y., Jeon, Y., Cho, N., Jang, G., and Bang, D. (2019). Lineage tracing using a Cas9-deaminase barcoding system targeting endogenous L1 elements. Nat. Commun. *10*, 1234. https://doi.org/10.1038/s41467-019-09203-z.

53. Zhao, D., Li, J., Li, S., Xin, X., Hu, M., Price, M.A., Rosser, S.J., Bi, C., and Zhang, X. (2021). Glycosylase base editors enable C-to-A and C-to-G base changes. Nat. Biotechnol. *39*, 35–40. https://doi.org/10.1038/s41587-020-0592-2.

54. Li, P., Su, Q., Xu, Y., He, J., Wang, L., Wang, Y., Li, H., Lan, K., Zheng, H., Zhu, D., et al. (2023). Multiplex Genome Editing of Human T Cells with Innovative Transformer Base Editor (tBE) for Construction of Next Generation CAR-T Therapies. Blood *142*, 6825. https://doi.org/10.1182/blood-2023-178504.

55. Weng, C., Yu, F., Yang, D., Poeschla, M., Liggett, L.A., Jones, M.G., Qiu, X., Wahlster, L., Caulier, A., Hussmann, J.A., et al. (2024). Deciphering cell states and genealogies of human haematopoiesis. Nature *627*, 389–398. https://doi.org/10.1038/s41586-024-07066-z.

56. Rusk, N. (2019). Mitochondrial barcodes. Nat. Methods *16*, 361. https://doi.org/10.1038/s41592-019-0416-9.

57. Zhang, L., Yu, X., Zheng, L., Zhang, Y., Li, Y., Fang, Q., Gao, R., Kang, B., Zhang, Q., Huang, J.Y., et al. (2018). Lineage tracking reveals dynamic relationships of T cells in colorectal cancer. Nature *564*, 268–272. https://doi.org/10.1038/s41586-018-0694-x.

58. Cao, G., Hu, Y., Pan, T., Tang, E., Asby, N., Althaus, T., Wan, J., Riedell, P.A., Bishop, M.R., Kline, J.P., and Huang, J. (2025). Two-stage CD8(+) CAR T-cell differentiation in patients with large B-cell lymphoma. Nat. Commun. *16*, 4205. https://doi.org/10.1038/s41467-025-59298-w.

59. Satija, R., Farrell, J.A., Gennert, D., Schier, A.F., and Regev, A. (2015). Spatial reconstruction of single-cell gene expression data. Nat. Biotechnol. *33*, 495–502. https://doi.org/10.1038/nbt.3192.

60. Wolf, F.A., Angerer, P., and Theis, F.J. (2018). SCANPY: large-scale single-cell gene expression data analysis. Genome Biol. *19*, 15. https://doi.org/10.1186/s13059-017-1382-0.

61. Saelens, W., Cannoodt, R., Todorov, H., and Saeys, Y. (2019). A comparison of single-cell trajectory inference methods. Nat. Biotechnol. *37*, 547–554. https://doi.org/10.1038/s41587-019-0071-9.

62. Street, K., Risso, D., Fletcher, R.B., Das, D., Ngai, J., Yosef, N., Purdom, E., and Dudoit, S. (2018). Slingshot: cell lineage and pseudotime inference for single-cell transcriptomics. BMC Genom. *19*, 477. https://doi.org/10.1186/s12864-018-4772-0.

63. Ji, Z., and Ji, H. (2016). TSCAN: Pseudo-time reconstruction and evaluation in single-cell RNA-seq analysis. Nucleic Acids Res. *44*, e117. https://doi.org/10.1093/nar/gkw430.

64. Qiu, X., Hill, A., Packer, J., Lin, D., Ma, Y.A., and Trapnell, C. (2017). Single-cell mRNA quantification and differential analysis with Census. Nat. Methods *14*, 309–315. https://doi.org/10.1038/nmeth.4150.

65. Li, L., Bowling, S., McGeary, S.E., Yu, Q., Lemke, B., Alcedo, K., Jia, Y., Liu, X., Ferreira, M., Klein, A.M., et al. (2023). A mouse model with high clonal barcode diversity for joint lineage, transcriptomic, and epigenomic profiling in single cells. Cell *186*, 5183–5199.e22. https://doi.org/10.1016/j.cell.2023.09.019.

66. Mao, S., Zhang, C., Chen, R., Tang, S., Fan, X., and Hu, J. (2025). Cell lineage tracing: Methods, applications, and challenges. Quant. Biol. *13*, e70006. https://doi.org/10.1002/qub2.70006.

67. Lange, M., Piran, Z., Klein, M., Spanjaard, B., Klein, D., Junker, J.P., Theis, F.J., and Nitzan, M. (2024). Mapping lineage-traced cells across time points with moslin. Genome Biol. *25*, 277. https://doi.org/10.1186/s13059-024-03422-4.

68. Loveless, T.B., Grotts, J.H., Schechter, M.W., Forouzmand, E., Carlson, C.K., Agahi, B.S., Liang, G., Ficht, M., Liu, B., Xie, X., and Liu, C.C. (2021). Lineage tracing and analog recording in mammalian cells by single-site DNA writing. Nat. Chem. Biol. *17*, 739–747. https://doi.org/10.1038/s41589-021-00769-8.

69. Ren, X., Yang, Z., Xu, J., Sun, J., Mao, D., Hu, Y., Yang, S.J., Qiao, H.H., Wang, X., Hu, Q., et al. (2014). Enhanced specificity and efficiency of the CRISPR/Cas9 system with optimized sgRNA parameters in Drosophila. Cell Rep. *9*, 1151–1162. https://doi.org/10.1016/j.celrep.2014.09.044.

70. Islam, M., Yang, Y., Simmons, A.J., Shah, V.M., Musale, K.P., Xu, Y., Tasneem, N., Chen, Z., Trinh, L.T., Molina, P., et al. (2024). Temporal recording of mammalian development and precancer. Nature *634*, 1187–1195. https://doi.org/10.1038/s41586-024-07954-4.

71. Liu, Z., Zeng, H., Xiang, H., Deng, S., and He, X. (2024). Achieving single-cell-resolution lineage tracing in zebrafish by continuous barcoding mutations during embryogenesis. J. Genet. Genomics *51*, 947–956. https://doi.org/10.1016/j.jgg.2024.04.004.

72. Pham, P., Afif, S.A., Shimoda, M., Maeda, K., Sakaguchi, N., Pedersen, L.C., and Goodman, M.F. (2017). Activation-induced deoxycytidine deaminase: Structural basis for favoring WRC hot motif specificities unique among APOBEC family members. DNA Repair *54*, 8–12. https://doi.org/10.1016/j.dnarep.2017.03.007.

73. Martin, A., and Scharff, M.D. (2002). Somatic hypermutation of the AID transgene in B and non-B cells. Proc. Natl. Acad. Sci. USA *99*, 12304–12308. https://doi.org/10.1073/pnas.192442899.

74. Hayal, T.B., Mulla, A.A., Allan, D.S.J., Duncan, B.B., Joshi, S., Hong, S.G., Basar, R., Rezvani, K., Childs, R.W., Wu, C., and Dunbar, C.E. (2025). Optimization of lentiviral delivery of barcoded anti-CD20 chimeric antigen receptors into rhesus macaque and human natural killer cells. Mol. Ther. Methods Clin. Dev. *33*, 101473. https://doi.org/10.1016/j.omtm.2025.101473.

75. Sheih, A., Voillet, V., Hanafi, L.A., DeBerg, H.A., Yajima, M., Hawkins, R., Gersuk, V., Riddell, S.R., Maloney, D.G., Wohlfahrt, M.E., et al. (2020). Clonal kinetics and single-cell transcriptional profiling of CAR-T cells in patients undergoing CD19 CAR-T immunotherapy. Nat. Commun. *11*, 219. https://doi.org/10.1038/s41467-019-13880-1.

76. Allen, M.E., Zhou, W., Thangaraj, J., Kyriakakis, P., Wu, Y., Huang, Z., Ho, P., Pan, Y., Limsakul, P., Xu, X., and Wang, Y. (2019). An AND-Gated Drug and Photoactivatable Cre-loxP System for Spatiotemporal Control in Cell-Based Therapeutics. ACS Synth. Biol. *8*, 2359–2371. https://doi.org/10.1021/acssynbio.9b00175.

77. Pei, W., Feyerabend, T.B., Rössler, J., Wang, X., Postrach, D., Busch, K., Rode, I., Klapproth, K., Dietlein, N., Quedenau, C., et al. (2017). Polylox barcoding reveals haematopoietic stem cell fates realized in vivo. Nature *548*, 456–460. https://doi.org/10.1038/nature23653.

78. Pei, W., Shang, F., Wang, X., Fanti, A.K., Greco, A., Busch, K., Klapproth, K., Zhang, Q., Quedenau, C., Sauer, S., et al. (2020). Resolving Fates and Single-Cell Transcriptomes of Hematopoietic Stem Cell Clones by PolyloxExpress Barcoding. Cell Stem Cell *27*, 383–395.e8. https://doi.org/10.1016/j.stem.2020.07.018.

79. Salla, M., Obermayer, B., Cotta, M., Friebel, E., Campo-Garcia, J., Charalambous, G., Bueno, R.J., Lieu, D., Dabek, P., Helmuth, A., et al. (2025). Cryo-mtscATAC-seq for single-cell mitochondrial DNA genotyping and clonal tracing in archived human tissues. bioRxiv, 2025.09.17.675534. https://doi.org/10.1101/2025.09.17.675534.

80. Anderson, N.D., Birch, J., Accogli, T., Criado, I., Khabirova, E., Parks, C., Wood, Y., Young, M.D., Porter, T., Richardson, R., et al. (2023). Transcriptional signatures associated with persisting CD19 CAR-T cells in children with leukemia. Nat. Med. *29*, 1700–1709. https://doi.org/10.1038/s41591-023-02415-3.

81. Rade, M., Fandrei, D., Kreuz, M., Seiffert, S., Grahnert, A., Friedrich, M., Wiemers, T., Born, P., Fischer, L., Weidner, H., et al. (2026). A longitudinal single-cell atlas to predict outcome and toxicity after BCMA-directed CAR T cell therapy in multiple myeloma. Cancer Cell *44*, 586–603.e9. https://doi.org/10.1016/j.ccell.2025.10.014.

82. Fang, C., Wang, L., Zhao, W., Wang, L., Huang, W., Chen, Z., Wang, Y., Tan, K., Guo, X., Xu, Y., et al. (2025). Integrated clinical and single-cell profiling of BCMA CAR-T therapy in relapsed/refractory multiple myeloma. J. Transl. Med. *23*, 1251. https://doi.org/10.1186/s12967-025-07106-w.

83. Wilson, T.L., Kim, H., Chou, C.H., Langfitt, D., Mettelman, R.C., Minervina, A.A., Allen, E.K., Métais, J.Y., Pogorelyy, M.V., Riberdy, J.M., et al. (2022). Common Trajectories of Highly Effective CD19-Specific CAR T Cells Identified by Endogenous T-cell Receptor Lineages. Cancer Discov. *12*, 2098–2119. https://doi.org/10.1158/2159-8290.Cd-21-1508.

84. Legut, M., Dolton, G., Mian, A.A., Ottmann, O.G., and Sewell, A.K. (2018). CRISPR-mediated TCR replacement generates superior anticancer transgenic T cells. Blood *131*, 311–322. https://doi.org/10.1182/blood-2017-05-787598.

85. Morton, L.T., Reijmers, R.M., Wouters, A.K., Kweekel, C., Remst, D.F.G., Pothast, C.R., Falkenburg, J.H.F., and Heemskerk, M.H.M. (2020). Simultaneous Deletion of Endogenous TCRαβ for TCR Gene Therapy Creates an Improved and Safe Cellular Therapeutic. Mol. Ther. *28*, 64–74. https://doi.org/10.1016/j.ymthe.2019.10.001.

86. Foy, S.P., Jacoby, K., Bota, D.A., Hunter, T., Pan, Z., Stawiski, E., Ma, Y., Lu, W., Peng, S., Wang, C.L., et al. (2023). Non-viral precision T cell receptor replacement for personalized cell therapy. Nature *615*, 687–696. https://doi.org/10.1038/s41586-022-05531-1.

87. Luo, X., Zhang, L., Li, Y., Li, C., Sun, G., Zhang, C., Fu, Y., Lv, H., Liu, M., Cui, H., et al. (2025). Full-Length Immune Repertoire Reconstruction and Profiling at the Transcriptome Level Using Long-Read Sequencing. Clin. Chem. *71*, 274–285. https://doi.org/10.1093/clinchem/hvae138.

88. Frede, J., Poller, J.C., Shi, K., Stuart, H., Sotudeh, N., Havig, C., Lim, K., Wiggers, C.R.M., Cho, E.Y., Vijaykumar, T., et al. (2025). The endogenous T cell landscape is reshaped by CAR-T cell therapy and predicts treatment response in multiple myeloma. Leukemia *39*, 3004–3014. https://doi.org/10.1038/s41375-025-02766-5.

89. Penter, L., Borji, M., Nagler, A., Lyu, H., Lu, W.S., Cieri, N., Maurer, K., Oliveira, G., Al'Khafaji, A.M., Garimella, K.V., et al. (2024). Integrative genotyping of cancer and immune phenotypes by long-read sequencing. Nat. Commun. *15*, 32. https://doi.org/10.1038/s41467-023-44137-7.

OMTA, Volume *34*

Supplemental information

The development of single-cell lineage tracing technology and its application in immunotherapy

Jun Wang, Zhisen Li, Jia Chen, Huan Li, Fei Chen, Lei Tan, Xiaoge Chen, Song Liu, Wenfeng Zhang, and Hongwei Shao

Table S1: Comparison of core characteristics across different cell lineage tracing barcode types

Type	Characteristics	Application	Method	Principle	Source Tagging	Testing Method	Advantages	Limitations	References
Exogenous engineered barcodes (Static)	Relies on the integration of synthetic DNA elements; mainly used for clone identification and early expansion analysis	Suitable for experiments requiring high controllability, such as developmental biology and tumor heterogeneity studies	Polylox barcode	Exogenous Cre recombinase mediates stochastic recombination between multiple loxP sites within a preset barcode cassette, generating unique heritable DNA rearrangements	Exogenously introduced loxP site arraysou	Identify rearranged sequences of the exogenous loxP array via singlecell sequencing (scRNAseq/snRNAseq)	Controllable labeling (inducible by light or drugs); high flexibility; generates high barcode diversity	Requires exogenous Cre expression; recombination efficiency is affected by loxP site configuration	Orban et al.41
	Uses transposons or lentiviruses to randomly integrate exogenous DNA fragments; integration sites serve as markers	Suitable for labeling stem cells, cancer cells, and experiments requiring high labeling diversity	Integration barcode	Transposons or lentiviruses randomly integrate exogenous DNA fragments into the host genome; the integration sites or fragments themselves serve as unique barcodes that are inherited through cell divisions	Exogenously integrated DNA fragments	Identify integrated DNA fragments via singlecell sequencing (scRNAseq/snRNAseq) or thirdgeneration sequencing	High marker diversity; applicable to stem cells and cancer cells	Viral integration may affect genomic stability; randomness can lead to uneven distribution of markers	Schmidt et al.42
Exogenous engineered barcodes (Dynamic)	Uses programmable nucleases to induce mutations at specific loci, enabling longterm lineage recording and fate branching analysis	Suitable for simultaneous lineage tracing and singlecell transcriptomics; construction of highresolution lineage trees	LINNAEUS	CRISPR/Cas9 induces insertions and deletions (InDels) at specific genomic loci; these “genetic scars” serve as barcodes read out by scRNAseq	InDels induced by CRISPR/Cas9	Analyze scar sequences generated by InDels using bulk RNAseq or scRNAseq	Singlecell resolution; compatible with scRNAseq; enables dynamic recording of cellular processes.	Editing efficiency may be limited by the number of available target sites	Spanjaard et al.43
	Similar to LINNAEUS, but built into an engineered mouse model that allows druginducible initiation of recording at any time point	Suitable for highresolution in vivo lineage tracing, especially when recording needs to be initiated at specific time points	CARLIN	An engineered mouse model harboring an array of gRNA targets and Teton inducible Cas9; doxycycline induces Cas9 to generate InDel mutations at the target array, producing transcriptionally compatible barcodes	InDels induced by CRISPR/Cas9	Analyze InDel scar sequences via bulk RNAseq or scRNAseq	Singlecell resolution; compatible with scRNAseq; dynamically inducible at desired time points	Requires preengineered mouse model; editing efficiency is influenced by the number of available targets	Bowling et al.44; Abdullah et al.45
	Uses a cytidine deaminase to introduce C→T substitutions in a barcode cassette, avoiding doublestrand breaks	Suitable for longterm, lowperturbation lineage recording	SMALT system	A cytidine deaminase (AID) introduces heritable C→T substitutions within an engineered barcode cassette, avoiding doublestrand breaks and reducing information loss	Base substitutions induced by cytidine deaminase	Identify base substitutions via scRNAseq or snRNAseq.	No doublestrand breaks (DSBs), advantageous for monitoring sensitive processes like embryogenesis; highdensity labeling	Relies on exogenous mRNA delivery; requires highthroughput sequencing analysis	Liu et al.46; Chen et al.47
	Uses a modified hgRNA to guide Cas9 to cut its own transcription site, accumulating mutations to form dynamic barcodes	Suitable for cumulative recording systems requiring extremely high barcode diversity	hgRNA/CRISPR	hgRNA guides Cas9 to cut its own transcription site; each cut generates InDels. Combining multiple hgRNA sites exponentially increases barcode diversity	InDels induced by CRISPR/Cas9	Analyze InDel mutations at hgRNA loci by sequencing	Extremely high barcode diversity; enables cumulative recording.	Depends on delivery and expression of the exogenous system	Kalhor et al.48
Endogenous geneticbarcode (Inducedbarcodes)	Uses base editors to introduce singlenucleotide variants without generating doublestrand breaks	Suitable for longterm lineage tracing, especially when avoiding DSBs is critical	Base editor barcode	Base editors (e.g., dCas9deaminase fusions) introduce precise singlenucleotide variants (SNVs) at target DNA sequences; accumulated mutations serve as heritable barcodes	SNVs induced by base editors	Identify base substitutions via singlecell sequencing	No DSBs, favorable for monitoring sensitive processes such as embryogenesis; highdensity labeling	Depends on delivery of exogenous base editors; requires highthroughput sequencing analysis	Hwang et al.49; Zhao et al.50; Li et al.51
	Utilizes the cell’s own genetic variation (e.g., mitochondrial DNA mutations) for lineage tracing without introducing exogenous sequences	Suitable for most cell types, especially primary cells sensitive to genetic manipulation and clinical samples	Mitochondrial DNA barcode	Uses the inherently high mutation rate of mitochondrial DNA (mtDNA) (10–100× that of nuclear DNA) to naturally accumulate mutations during cell proliferation, forming genetic markers	Natural mutations in mtDNA as endogenous biomarkers	Capture mtDNA sequences and detect mutations via singlecell sequencing (e.g., scRNAseq, scATACseq)	Noninvasive, no gene editing required; suitable for primary cells, especially T cells	mtDNA copy number variation can affect marker reliability; applicability may be limited across different tissue types.	Weng et al.52; Rusk53; Lareau et al.54; Ludwig et al.55
Endogenous geneticbarcode (Naturalbarcodes）	Uses the vast diversity generated by Tcell receptor (TCR) gene rearrangement as a clonal marker, especially suitable for Tcell lineage tracing	Applicable to Tcell lineage tracing; used to study Tcell clonal evolution, tumor microenvironment, and treatment efficacy	TCR barcode	TCR generates an extremely diverse repertoire through somatic recombination. Each T cell and its progeny carry a unique TCR sequence that serves as a lineage marker	TCR gene sequences as natural barcodes	Identify TCR sequences via singlecell TCR sequencing (scTCRseq) or combined scRNAseq	Noninvasive, no gene editing required; suitable for primary T cells; directly reflects Tcell clonal dynamics	TCR sequences are highly heterogeneous with minimal sharing between patients—highly personalized; can only track Tcell clones, not reconstruct complete developmental lineages	Zhang et al.56; Cao et al.57

Table S2. Summary of Single-Cell Lineage Tracing and Trajectory Inference Methods

Method	Main principle	Main use	Reference
Monocle	Orders cells along pseudotime based on transcriptional similarity	Reconstruction of developmental trajectories	Qiu et al. 63
Slingshot	Infers lineage structure using cluster-based minimum spanning trees and simultaneous principal curves	Branching trajectory inference	Street et al.61
TSCAN	Uses clustering and minimum spanning tree-based ordering	Pseudotime analysis	Ji and Ji.62

Table S3: Detailed comparison and future perspectives of singlecell lineage tracing (SCLT) strategies

Strategy	Barcode Type	More Suitable Application Scope	Experimental Complexity	Integration with scRNA-seq	Already Used for T Cell / CAR-T Lineage Tracing	Main Advantages	Main Limitations	Latest Progress and Prospects	References
TCR barcode	Endogenous, clonotype-based	Most directly applicable to T cells/engineered T cells; short- to mid-term clonal expansion, persistence, state differentiation	Moderate	Strong; scRNA + paired TCR-seq is mature	T cells: widely used; CAR-T: clearly used for in vivo clonal dynamics studies in patients	Natural, no labeling required; directly corresponds to antigen recognition history; most suitable for T cell studies	Can only track clones after V(D)J rearrangement; cannot reveal earlier ancestors or intra-clonotype hierarchy	Moving from "clonal counting" toward clonal fate rules and longitudinal efficacy association; one of the most practical barcodes for clinical engineered Tcell studies	Zhang et al.57; Cao et al.58
mtDNA barcode	Endogenous, somatic mutation-based	Short-term tracking of primary cells; clinical cohorts and pathology samples (more accessible than most prospective engineered barcodes)	High (limited by sequencing depth and platform type)	Achieved (e.g., integrated with spatial transcriptomics)	T cells: proven feasible; CAR-T: currently nonmainstream	No exogenous gene introduction; high usability in clinical samples	Limited by mitochondrial inheritance patterns and tissue applicability; detection constrained by sequencing depth/platform; mtDNA variants not strictly neutral in all contexts	Developed to work with frozen samples and spatial transcriptomics for lineage tracing in intact human tissues	Ludwig et al.55; Lareau et al.54; Salla et al.80; Ludwig et al.81
Integration barcode	Exogenous, insertion-based	Immune cell expansion and fate analysis; research models, cell product evaluation, preclinical validation	Moderate (library construction and viral packaging required)	Integrated (e.g., LARRY)	CAR-T: used for clonal dynamics analysis of products (combined with integration site analysis)	Relatively simple to construct; can label large numbers of cells at once	Random integration may affect host gene regulation or cell fitness; insertion site safety must be carefully assessed	Needs optimization for safety and clinical translation; LARRY links scRNAseq state map to cell fate map	Schmidt et al.45; Weinreb et al.74; Hayal et al.75; Sheih et al.76
Polylox barcode	Exogenous, DNA recombination-based (Cre-loxP)	Precise in vivo barcode generation; long-term in vivo tracking; conditional gene switches/circuit control; prebuilt in transgenic mice (not directly applicable to patients)	High (requires Cre-loxP system and transgenic mouse models)	Achieved (PolyloxExpress allows simultaneous barcode + transcriptome reading)	T cells: rarely used, not a mainstream strategy	Does not depend on cellular transcriptional expression; suitable for long-term in vivo tracking; enables high-resolution endogenous labeling in vivo	Barcode diversity determined by preset cassette structure; cannot be directly applied to patients	Requires further development for use in T cells; already applied to HSC fate mapping	Orban et al.44; Pei et al.78; Pei et al.79
SMALT (substitution-based)	Exogenous, basesubstitution (AID)	Tracking cell division and clonal expansion history (e.g., addressing whether CART expansion is dominated by few clones or many)	High (requires expression of AID, etc.)	Potential, but no mature solution mentioned	Not explicitly mentioned, but inferred to be applicable for CART clonal expansion studies	Less prone to information loss from large deletions; better for long-term recording; substitution patterns resemble SNP data, allowing use of phylogenetic frameworks	Not uniformly random; sequencecontext bias (e.g., WRC hotspots); risk of convergent edits and homoplasy; writing rate not an intrinsic clock	Has potential to resolve postinfusion CART clonal dominance and longterm persistence	Liu et al.46; Chen et al.47; Pham et al.72; Martin and Scharff73
Base editor barcode	Exogenous, baseediting (nCas9/deaminase)	Similar to SMALT, suitable for continuous mutation recording	High (requires nCas9/deaminase system)	Challenging; requires deep sequencing and rational barcode design	Not explicitly mentioned; early stage	Does not rely on doublestrand breaks; avoids large deletions and target collapse; allows continuous substitution mutations	Writing rate not synchronized with mitosis; not fully random; influenced by editable motif distribution, deaminase sequence bias, and narrow editing window; immature technology	Needs further research and optimization; Transformer Base Editor achieves up to 90% editing efficiency in primary human T cells	Hwang et al.49; Zhao et al.50; Li et al.51
LINNAEUS (CRISPR scar)	Exogenous, CRISPR indelbased	Prospective, hierarchical preclinical studies (reconstructs lineage trees but rarely captures all division events)	High (requires transgenic reporter sites and inducible Cas9)	Integrated (scar barcodes read out by scRNAseq)	Has potential, but limited by editingassociated toxicity and impact on T cell function	Inducible, prospective, controllable	Scar generation not directly linked to mitosis; barcode generation stops early, leading to incomplete coverage of terminal branches; indel bias, homoplasy, dropout, etc.	Dropout can be corrected by introducing "barcode+UMI+scar sequence"; methods like moslin integrate lineage constraints into state alignment	Spanjaard et al.41; Mao et al.65; Lange et al.66
CARLIN / DARLIN	Exogenous, CRISPR indelbased (DARLIN adds TDT)	Prospective, hierarchical preclinical studies; DARLIN suitable for applications requiring high barcode diversity (e.g., in mice)	Very high (requires engineered mouse models or complex constructs)	Integrated (DARLIN enables multiomics like Camelliaseq)	Mostly limited to mouse models; CARLIN used for lineage tracing of antigenspecific CD8 T cell clones	Inducible, generalizable; DARLIN has larger barcode capacity and diversity (via TDT); supports multiomics	Same issues as other indel barcodes (bias, homoplasy, dropout, low recovery rates); editingassociated genotoxicity, offtarget effects; potential impact on T cell fitness	Camelliaseq enables fourmodal singlecell profiling (barcode, transcriptome, chromatin accessibility, DNA methylation) but requires deep coverage and is costly	Bowling +A1:J10et al.42; Abdullah et al.43; Li et al.67
